# In-Phase Bilateral Upper Limb Exercises Improve Cognitive and Motor Functions in Progressive Multiple Sclerosis: A Pilot Randomized Controlled Trial

**DOI:** 10.3390/brainsci16020191

**Published:** 2026-02-05

**Authors:** Dimitris Sokratous, Charalambos Costa Charalambous, Marios Pantzaris, Kyriaki Michailidou, Nikos Konstantinou

**Affiliations:** 1 Physiotherapy Unit, Neurology Clinics, The Cyprus Institute of Neurology and Genetics, Nicosia 2371, Cyprus; 2 Department of Rehabilitation Sciences, Faculty of Health Sciences, Cyprus University of Technology, Limassol 3036, Cyprus; nikos.konstantinou@cut.ac.cy; 3 Department of Neurology, School of Medicine, Duke University, Durham, NC 27710, USA; cccharalam@gmail.com; 4 Neuroimmunology Department, The Cyprus Institute of Neurology and Genetics, Nicosia 2371, Cyprus; pantzari@cing.ac.cy; 5 Biostatistics Department, The Cyprus Institute of Neurology and Genetics, Nicosia 2371, Cyprus; kyriakimi@cing.ac.cy

**Keywords:** multiple sclerosis, bilateral exercise, cognitive processing, motor function, quality of life

## Abstract

**Highlights:**

**What are the main findings?**

• In-phase bilateral upper limb exercises significantly improved information processing speed in progressive multiple sclerosis.
• The intervention also enhanced motor function, reduced fatigue, and improved quality of life.

**What are the implications of the main findings?**

• In-phase bilateral exercises offer a simple, low cognitive-demand approach to cognitive–motor rehabilitation in progressive multiple sclerosis.
• This exercise strategy can be easily integrated into clinical and group-based rehabilitation programs.

**Abstract:**

**Background and Purpose:** Progressive multiple sclerosis impairs cognitive and motor functions and reduces quality of life. Complex goal-directed movements are challenging due to cognitive deficits, whereas in-phase bilateral exercises require less attentional demand and cognitive effort. This type of exercise may therefore improve both cognitive and motor functions. Previous studies in people with progressive multiple sclerosis suggested a strong association between cognitive and upper limb functions; however, the effects of in-phase bilateral exercises remain unclear. **Objectives:** To evaluate the effects of in-phase bilateral upper limb exercise on cognitive processing, motor functions, and quality of life in people with progressive multiple sclerosis. **Methods:** Twenty participants (11 females, mean age = 55.8 years) were randomized (1:1) to an experimental or active control group for a 12-week intervention. The experimental group performed in-phase bilateral upper limb exercises while the active control group performed conventional exercises. ANOVA was conducted to determine the effect of intervention on information processing speed, motor function, fatigue, and quality of life. **Results:** Post hoc analyses revealed that the experimental group demonstrated significantly greater improvements than the active control group in information processing speed (*t*(18) = 8.6, *p* < 0.05), as well as across all exploratory secondary outcome measures (all *p* < 0.05). **Conclusions:** This pilot randomized controlled trial suggests that in-phase bilateral exercises, which demand less cognitive effort than other forms of bilateral coordination, are associated with improvements in information processing speed, motor functions, fatigue, and quality of life in people with progressive multiple sclerosis.

## 1. Introduction

Multiple sclerosis (MS) is the most common inflammatory and neurodegenerative disease of the central nervous system [1]. Given that most people with Relapsing-Remitting Multiple Sclerosis eventually transit to Progressive MS (PMS), which includes both Secondary Progressive MS and Primary Progressive MS [1], understanding the progression and treatment options for PMS is of critical importance. This transition is connected with an increase in disability and a steady accumulation of neurological impairment.

Not only do people with PMS (pwPMS) experience physical impairment but they also usually suffer from cognitive dysfunction [2], which significantly affects their quality of life (QoL). Among cognitive domains, information processing speed is commonly impaired [3,4] in both Secondary and Primary Progressive MS [5]. Various cognitive rehabilitation programs [4,6] have shown efficacy in improving MS-related cognitive dysfunctions, such as learning, memory, attention, and cognitive processing.

Beyond cognitive training, exercise has emerged as a promising multimodal intervention, as it has shown to improve both cognitive and motor functions [7,8], as well as QoL in people with MS [9], offering a holistic approach compared to cognitive training alone [10]. Evidence from healthy individuals and people with MS indicated a close relationship between cognitive function and upper limb performance [11,12], supported by dense neural projections linking the anterior cingulate cortex, motor cortex, and spinal cord pathways [13]. Within this framework, impairments in information processing speed have been shown to negatively affect manual dexterity, thereby compromising functional independence and performance in activities of daily living [12,14].

Emerging evidence indicates a close interdependence between cognitive function and motor control [13,15,16]. Within this neurofunctional framework, bilateral upper limb movements, particularly in-phase bilateral movements where both limbs move simultaneously in the same direction, have emerged as a promising intervention for enhancing cognitive performance. These movements engage both hemispheres, promoting interhemispheric communication via the corpus callosum and strengthening connectivity between the supplementary motor area and the primary motor cortex [13,17,18]. These neural adaptations facilitate a more efficient integration of sensory and motor information, leading to improvements in information processing speed, a key determinant of higher-order cognitive functions such as attention, working memory, and executive control [19,20,21,22,23].

Importantly, in-phase bilateral movements require lower attentional and motor control demands than anti-phase or unilateral patterns, making them particularly suitable for rehabilitation in populations with cognitive or motor impairments, such as pwPMS [24,25,26]. Preliminary evidence from a clinical trial in people with Relapsing-Remitting MS [27] has shown that in-phase bilateral upper limb exercises significantly improve information processing speed, further supporting its potential as a targeted intervention to enhance both cognitive and motor outcomes in PMS.

The cognitive benefits of exercise may be further enhanced through group-based circuit training, which integrates physical, cognitive, and social components. Group exercise not only provides structured physical conditioning but also fosters social interaction, enhancing motivation and engagement while activating brain regions involved in social cognition and executive control [28,29]. The interactive nature of group training creates an engaging environment that promotes adherence and exercise intensity, both key determinants of cognitive improvement [30]. Moreover, structured group exercise programs, particularly those implemented in circuit formats, have been connected with greater neuroplasticity, improved brain function [29], and reduced risk of cognitive decline [28]. Circuit training, characterized by rapid transitions between different exercises, delivers simultaneous physical and cognitive stimulation. Within this framework, the motor–cognitive model proposed by Herold et al. (2018) suggests that embedding cognitive demands, such as task switching, attentional control and working memory, into movement optimally engages prefrontal and cerebellar networks, thereby enhancing executive functions [31].

Despite evidence for dual-task motor and cognitive training in pwPMS [7,32], it remains uncertain whether specific low-cognitive-demand exercise modalities can improve cognitive and motor functions comparably. This is particularly relevant for patients with cognitive impairments who may struggle to follow complex dual-task instructions. The present pilot study therefore implemented a structured, group-based circuit training intervention incorporating in-phase bilateral upper limb exercises designed to enhance information processing speed, manual dexterity, gait, balance, fatigue, and QoL in pwPMS.

## 2. Materials and Methods

**Figure 1:**
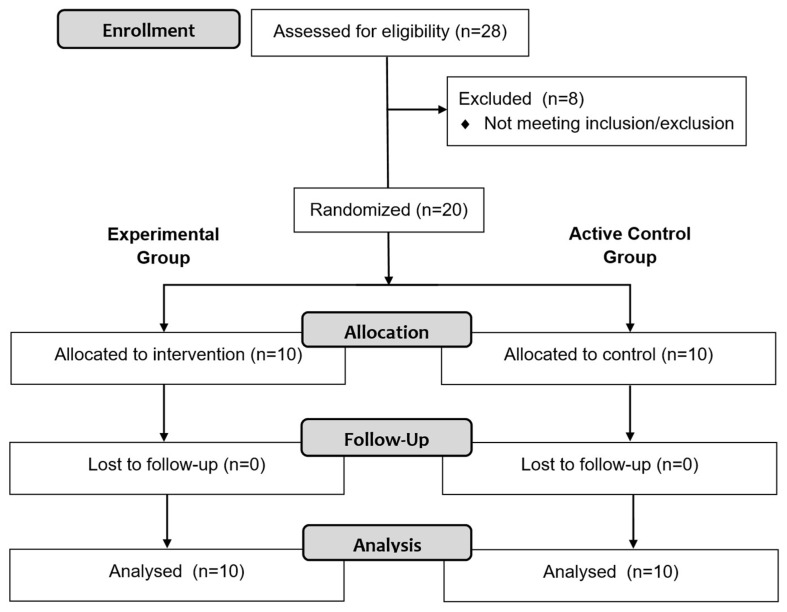
CONSORT flow diagram of study participants. Eight participants did not meet the inclusion criteria; therefore, they were not enrolled in the study. Enrolled participants were randomized to experimental (n = 10) and active control (n = 10) groups. All participants completed the study and none of them were excluded from data analysis.

Participants were recruited from The Cyprus Institute of Neurology and Genetics in September 2023. All participants underwent a neurological evaluation, and their individual medical records were reviewed to confirm eligibility prior to enrollment. A total of (n = 28) pwPMS provided informed consent; however, (n = 8) did not meet the eligibility criteria and were excluded (Figure 1), resulting in a final sample of (n = 20) participants. Participant allocation was completed between 25 September and 30 September 2023. Only the neurologist had access to identify the data and was blinded to group assignment. The study was conducted between 2 October and 22 December 2023, and was registered at ClinicalTrials.gov (NCT06436131). Ethical approval was obtained from the Cyprus National Bioethics Committee (EEBK/EΠ/2022/32).

The inclusion criteria included (1) diagnosis of PMS (Primary or/and Secondary PMS), (2) Expanded Disability Status Scale (EDSS) [33] score between three and six, (3) no relapse within the last 30 days, (4) aged between 30 and 70 years, and (5) Mini Mental State Examination (MMSE) [34] score between 20 and 30 (mild to no cognitive impairment). The exclusion criteria included (1) history of any disease affecting the central nervous system other than MS (e.g., stroke), (2) history of cardiovascular disease (e.g., myocardial infarction), (3) severe orthopedic disorders (e.g., knee/hip replacement), (4) mental disorders (e.g., depression), (5) pregnancy during the implementation of the study timeline, (6) hearing impairments (i.e., deafness), (7) visual deficit (e.g., optic neuritis), (8) history of epileptic seizures, and (9) spasticity level on upper or lower limbs more than 1+ (slight increase in muscle tone) according to the Modified Ashworth Scale [35].

Additionally, participants were advised to continue with their usual prescribed medication throughout the study, and they were advised to continue their usual routine and avoid receiving any other exercise programs during the study. They were also instructed to inform the researchers of any changes in medication or daily activities. Furthermore, all participants read and signed written informed consent, while all procedures were approved and conducted in accordance with the ethical guidelines of the Cyprus National Bioethics Committee before recruitment.

### 2.1. Study Design

**Figure 2:**
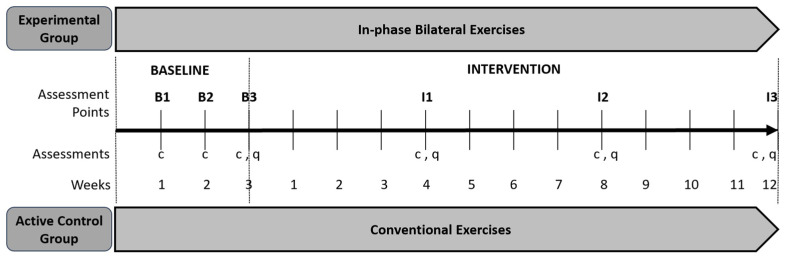
Timeline and schematic representation of the study design. B, baseline; I, intervention; c, clinical assessments; q, questionnaires. The two gray rows represent the experimental and the active control groups. The study spanned a total of 15 consecutive weeks, comprising of a 3-week baseline phase followed by a 12-week intervention phase. During the intervention phase, participants in the experimental group engaged in an exercise protocol emphasizing in-phase bilateral upper limb movements. In contrast, participants in the active control group completed a conventional exercise regimen.

The present study was an assessor-blinded, two-arm randomized controlled trial designed to evaluate the effects of a 12-week in-phase bilateral upper limb exercise protocol on information processing speed in pwPMS. Participants were randomly assigned to either an experimental (n = 10) or an active control group (n = 10) (Figure 2).

The experimental group received supervised training from a certified fitness instructor, whereas the active control group was supervised by a physiotherapist, both blinded to group allocation. Clinical assessments for both groups were conducted by an independent physiotherapist blinded to group allocation. To minimize intergroup contamination, training sessions for the two study groups were conducted separately. Randomization was conducted using computer-generated randomization, stratified by the EDSS score, age, gender, and hand dominance (Table 1), factors known to influence exercise performance [36,37,38] and relevant to the study’s outcome measures.

#### 2.1.1. Baseline Phase

All participants entered a three-week baseline phase that began simultaneously for the entire cohort, with assessments conducted once per week. Cognitive and motor outcomes were recorded at each time point, and two subjective questionnaires (see outcome measures) were administered during the final week of the baseline phase (Figure 2).

#### 2.1.2. Intervention Phase

The 12-week intervention included three sessions per week for the experimental group and one session per week for the active control group. The active control group exercised once weekly, a frequency selected to reflect standard physiotherapy practice recommended by physiotherapists within the national health system for chronic management of these conditions and for the maintenance of general health and QoL. For ethical reasons, a completely inactive control group was not included. This design allowed us to examine whether the specific exercise protocol conferred additional benefits beyond those expected from standard guideline-based activity levels.

#### 2.1.3. Experimental Grοup

Each intervention session consisted of three phases: a five-minute warm-up, 40–50 min of the main exercise protocol, and a five-minute cool-down. The warm-up included whole body range of motion exercises, while the cool-down comprised passive stretching of the muscle groups engaged during the main session.

Throughout the intervention period, each participant underwent three clinical assessments and completed two subjective questionnaires, resulting in three distinct data collection time points (Figure 2). In order to be included in the final analysis, participants were required to attend at least 27 of the 36 sessions (i.e., ≥75%) allocated to their study group [27,39].

#### 2.1.4. Main Exercise Protocol

Exercises were conducted in a sports hall and consisted of in-phase bilateral upper limb movements performed in a group-based circuit training format, adapted from MS guidelines [40] and previous studies in Relapsing-Remitting MS [27].

Each session included:

• Three sets of nine upper limb exercises targeting large muscle groups.
  (shoulder flexors, extensors, rotators, abductors, adductors, horizontal abductors/adductors; elbow flexors and extensors)
• Three lower limb exercises targeting large muscle groups.
  (hip flexors, extensors, abductors, adductors; knee and ankle flexors and extensors)

Lower limb exercises were interspersed between upper limb exercises to allow for muscle recovery. Each exercise lasted one minute, during which participants performed as many repetitions as possible. A two-minute rest period was provided between sets.

#### 2.1.5. Exercise Modalities and Progression

The program incorporated sports-based technical skills, including basketball (e.g., passing, catching, throwing) and volleyball (e.g., passing and receiving). Fitness exercises included diagonal movement patterns based on a proprioceptive neuromuscular facilitation technique [41] using resistance bands, as well as open-chain upper limb exercises performed with 1 kg dumbbells (e.g., shoulder flexion/extension and abduction/adduction with extended elbows) (Table 2).

To maintain participant engagement and ensure that the Rate of Perceived Exertion remained within the target range of three to six, the exercise protocol was progressively adapted over the 12-week intervention period. Progression was achieved through the use of elastic bands with varying resistance levels, along with the introduction of dumbbells with different weights and adjustments to passing distances during ball-based exercises.

All sessions were conducted in a temperature-controlled environment (24 °C) under standardized safety conditions (e.g., use of mats) to minimize the risk of falls. This approach ensured that exercises remained challenging yet achievable, maximizing training benefits while minimizing the risk of overexertion.

#### 2.1.6. Active Control Group

All exercise sessions and clinical assessments were conducted at the Physiotherapy Unit of the Cyprus Institute of Neurology and Genetics. Participants in the active control group attended one-on-one sessions with the same physiotherapist. Participants were required to complete at least 9 out of 12 sessions (i.e., 75%) for their data to be included in the analysis [27,39]. Every session consisted of a five-minute warm-up (i.e., whole body range of motion exercises), followed by 40–50 min of the main conventional exercise program described below and a cool-down for five minutes (i.e., passive stretching exercises of the muscle groups which are involved in the main part).

The conventional program was implemented according to previously published protocols and consisted of strengthening exercises for the major trunk muscle groups [42], resistance exercises for the upper limbs, and treadmill training [43].

##### Trunk Strengthening Exercises

The trunk-strengthening component consisted of exercises targeting the flexor (rectus abdominis) and extensor (erector spinae) muscles. During each exercise, participants held the final static position for three seconds and performed five to ten repetitions, depending on individual tolerance. A three-second pause was allowed between repetitions, with a maximum of one-minute rest between exercises. The difficulty level increased gradually by extending the hold time and increasing the number of repetitions, based on participants’ tolerance.

##### Upper Limb-Strengthening Exercises

The upper limb-strengthening component included exercises targeting the flexor, extensor, and internal and external rotator muscle groups of the shoulder joint, as well as the flexor and extensor muscle groups of the elbow joint. Each exercise was performed for five to ten repetitions and one to three sets, depending on individual tolerance. A three-second pause was allowed between repetitions, and a maximum of one-minute rest was provided between sets. The level of the difficulty was gradually increased by either increasing the number of repetitions or the resistance of the elastic band, based on participants’ capabilities.

##### Aerobic Exercises

To complete the session, all participants performed 10–15 min of treadmill walking at a pace of 1.5–2.5 km/h or cycling on a static cycle ergometer (MOTOmed Loop Parkinson) at 20–40 bpm. The intensity of these aerobic exercises was adjusted to individual tolerance.

### 2.2. Outcome Measures

Previous studies indicated that information processing speed is the most common cognitive deficit in pwPMS [3,4] and it is correlated with manual dexterity [14,44]. Therefore, this pilot clinical trial examined whether a specific exercise protocol, based on in-phase bilateral upper limb exercises, led to greater improvement compared to the minimum exercise recommendation of the national health system.

Cognitive processing and manual dexterity are closely intertwined, with one influencing the other. Several tasks requiring manual dexterity often engage cognitive processes, such as attention, memory, and problem-solving, suggesting a bidirectional relationship. This connection is particularly relevant to the current study, as our exercise protocol includes in-phase bilateral upper limb exercises. By targeting these motor skills, we anticipate an improvement in information processing speed, as the two are closely connected. Consequently, we have chosen information processing speed as our primary outcome measure, expecting it to be directly associated with the effects on manual dexterity, achieved through the in-phase bilateral upper limb exercise protocol.

To ensure methodological consistency, the same physiotherapist collected all data by performing the same methodological procedures in a quiet room, across all participants and across all time points, for both the experimental and active control groups. To prevent any decline in performance due to participant fatigue, all assessments were conducted between 9 a.m. and 11 a.m. in the same sequence as described below.

#### 2.2.1. Primary Outcome Measure

The primary outcome measure was information processing speed, assessed using the Symbol Digit Modalities Test (SDMT), a widely used and validated tool in people with MS [3]. The oral version of the SDMT was employed, in which participants were provided with a sheet displaying nine symbols, each paired with a corresponding number at the top of the page, referred to as the “key”. For example, the symbol “O” might be paired with the number “6”, so the correct response would be “six”. The remainder of the page contained a randomized sequence of these symbols. Participants were instructed to verbally identify the number corresponding to each symbol as quickly and accurately as possible over a two-minute period. The total score was calculated by subtracting the number of errors from the number of items completed. To minimize practice effects across multiple assessments, six alternate forms of the SDMT were created, one for each assessment point, with the order of symbols and corresponding key numbers rearranged [45]. To improve the stability of the study data, three SDMT assessments were conducted one week apart during the baseline phase and three assessments at four-week intervals during the intervention phase. Given the known day-to-day fluctuations in cognitive performance in pwPMS, averaging both baseline and intervention scores was intended to provide a more robust estimate of true performance and to reduce the influence of transient factors or outlier values.

#### 2.2.2. Secondary Outcome Measures

Exploratory secondary outcomes included manual dexterity, which was evaluated with the Purdue Pegboard Test (PPT) [46]; changes in gait speed were assessed by the Timed 25-Foot Walk Test [47], while walking ability, balance, and lower limb coordination were evaluated by the Six Spot Step Test [48]. Considering that fatigue and QoL are key factors affecting people with MS, additional exploratory secondary outcomes were also assessed. These included the Modified Fatigue Impact Scale, a subjective questionnaire assessing the effects of fatigue [49], and the Medical Outcomes Study Questionnaire Short Form 36 Health Survey, a tool for evaluating health-related QoL [50].

##### Medical Outcomes Study Questionnaire Short Form 36 Health Survey

This is a set of generic, coherent, and easily administered QoL subjective questionnaires [51]. There are 11 questions in the specific questionnaire administered by the assessor, with 36 items in total covering eight domains scaled from 0 to 100. Higher values indicate better health status. The eight domains include general health, vitality, physical function, role physical, bodily pain, role emotional, social functioning, and mental health. Participants needed between five to ten minutes to complete the questionnaire.

##### Modified Fatigue Impact Scale

It is a subjective questionnaire describing the effects of fatigue during the past four weeks [49]. The Modified Fatigue Impact Scale consists of 21 questions, rated from “0” (low rate) to “4” (high rate), and it is divided into three subscales (i.e., physical, cognitive, and psychosocial). The assessor records the total score of the test as the final test result. A higher score indicates greater impact of fatigue in an individual’s daily life.

##### Purdue Pegboard Test

This is a standardized test of manual dexterity [46]. It consists of four subtests, performed on a board in which pins, washers, and collars are placed by the participants into two parallel columns of holes, according to the subtest task. The first two subtests are unimanual tasks, which measure the dexterity of the right and left hands, respectively. The third subtest is a synchronous bimanual task that requires simultaneous use of both hands to grasp and place the pins. In the fourth subtest, participants perform alternating movements of both hands to complete assemblies of different types of pegs. The score is calculated based on the number of pegs inserted in 30 s for the first three subtests, and in 1 min for the fourth subtest.

##### Timed 25-Foot Walk

It is a quantitative assessment for mobility and lower limb function [47]. Participants are directed to one end of a marked 25-foot path and are instructed to walk as quickly as possible. Time is recorded the moment the participants lift a foot to start and ends when participants reach the 25-foot mark. The same task is immediately run again by having the participants walk back the same distance. As our participants may be using assistive devices for walking, they were instructed to use them for safety reasons. The final score was the mean score from the two completed trials.

##### Six Spot Step Test

It is a measure replicating a complex range of sensorimotor functions, such as lower limb strength, spasticity, coordination, and dynamic balance [48]. It is a timed walking test that involves kicking over several targets placed along a 5 m path. The specific test is cognitive demanding and also includes coordination and dynamic balance. The final score was the mean time of the four runs [52].

### 2.3. Analysis Plan

Within-group analysis: For each outcome measure, we calculated the mean values from each time point (separately for baseline and intervention). To investigate the effect of the exercise protocol on each of the outcome measures, separately for each group, we calculated the differences between the phases’ mean values. These differences reflect the degree of the intervention-elicited change on the clinical condition of all participants.

Between-groups analysis: To detect if there is a significant effect between the two study groups, comparisons were made between the difference in improvement between the two groups for each outcome measure. The difference in improvement from each group was calculated by the difference between baseline and intervention mean values of each outcome variable.

#### Statistical Analysis

Normality and sphericity assumptions were assessed, and appropriate adjustments were applied when violations were detected. When assumptions were not met, non-parametric Wilcoxon signed-rank tests with Greenhouse–Geisser correction were used; otherwise, change scores (Intervention − Baseline) were compared between groups using repeated-measures ANOVA. Significant effects were explored using Bonferroni-corrected post hoc *t*-tests. Associations between SDMT and PPT scores during the intervention period were examined using Pearson correlation coefficients, with significance thresholds adjusted for multiple testing (0.05/number of tests). As this was a pilot study, analyses were exploratory and focused on estimating effect sizes; therefore, no power analysis was conducted. The null hypothesis of no between-group differences in outcome changes was evaluated using an adjusted significance level of *p* < 0.05. All analyses were conducted in JASP version 0.19.1; https://jasp-stats.org (accessed on 12 February 2024).

## 3. Results

Following our sampling plan, a total of (n = 20) participants were enrolled and allocated to the experimental (n = 10) and the active control (n = 10) groups. All participants completed the exercise program without complaints or side effects. Additionally, the physician who evaluated participants for eligibility reviewed their individual medical records and confirmed that no changes occurred in medication during the study period. As described in detail below, the results indicated that participants from the experimental group had on average greater improvement when compared with the active control group on all outcome measures. Data are available at FIGSHARE: https://doi.org/10.6084/m9.figshare.26953714.v1, accessed on 9 August 2024.

### 3.1. Primary Outcome Measure—SDMT

**Figure 3:**
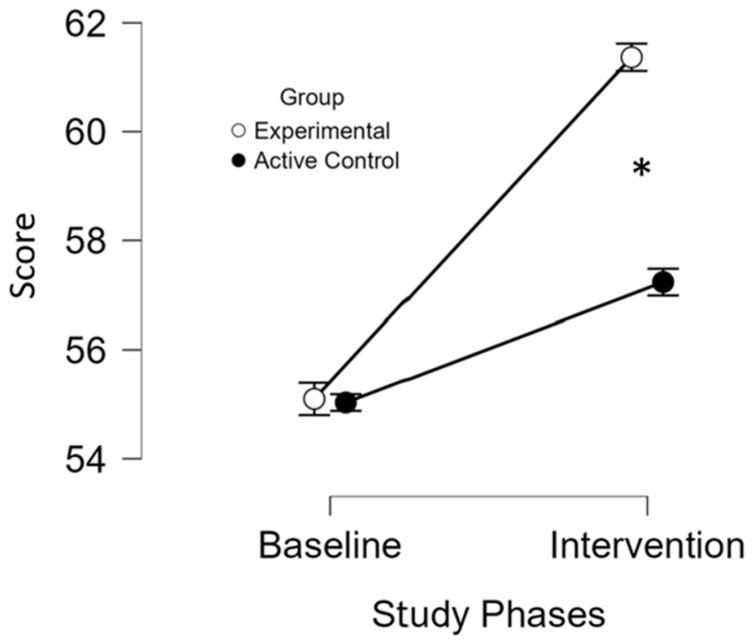
Symbol Digit Modalities Test performance across study phases and groups. *, significant improvement (*p* ≤ 0.05) at the specified assessment point between the two study groups. The x-axis represents the study phases, while the y-axis displays the mean test scores. Both groups began with comparable baseline values (*p* = 1), thereby reducing the likelihood that initial differences influenced the observed outcomes. Although both groups showed improvements over time, the experimental group (white circles) demonstrated a significantly greater increase in performance compared to the active control group (black circles) (*p* < 0.05). These findings suggest that the in-phase bilateral upper limb exercise protocol may enhance cognitive processing more effectively than the control intervention.

An increase in total scores and correct responses in SDMT indicates improvement of information processing speed. Both groups’ individual results (Table S1) and ANOVA analyses (Tables S11–S14) showed significant improvements from baseline to intervention (*p* < 0.001). The experimental group improved from mean (M) = 55.1, standard deviation (SD) = 13.8 to Μ = 61.3 (SD = 14.1), while the active control group increased from M = 55 (SD = 13.4) to M = 57.2 (SD = 13.2). A significant interaction between study phases and group (*p* < 0.001) indicated greater improvement in the experimental group. Baseline scores between study groups did not differ significantly (Figure 3), confirming their comparability. Post hoc Bonferroni analysis and *t*-test confirmed significantly greater improvements (*t*(18) = 8.6, *p* < 0.05) in the experimental group (M = 6.2 (SD = 1.4)) compared to the active control group (M = 2.2 (SD = 0.6)).

Focused on the primary outcome (i.e., SDMT), with an estimated effect size of partial η^2^ = 0.807, a significance level of α = 0.05, and a sample size of 20 participants, post hoc analysis indicated that the study had 100% statistical power to detect associations (1 − β = 1). The power analysis was conducted using G*Power3.1.

These findings suggest that the in-phase bilateral upper limb exercise protocol may enhance cognitive processing more effectively than the conventional exercises.

### 3.2. Secondary Outcome Measures

#### 3.2.1. Medical Outcomes Study Questionnaire Short Form 36 Health Survey

**Figure 4:**
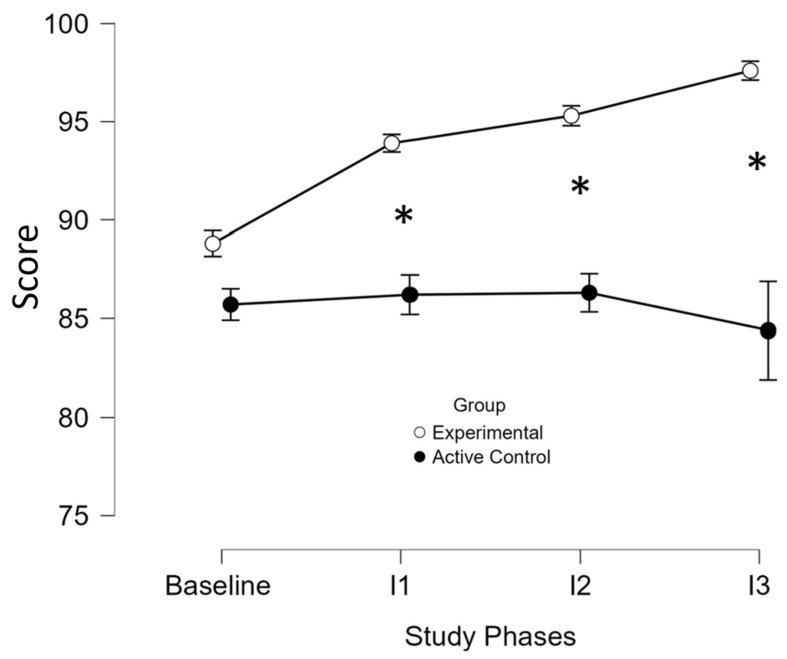
Performance of Medical Outcomes Study Questionnaire Short Form 36-Item Health Survey across study phases and groups. I, intervention; *, significant improvement (*p* ≤ 0.05) at the specified assessment point between the two study groups. The x-axis displays the assessment time points, including baseline (mean score) and three subsequent intervention phases (I1, I2, I3), while the y-axis represents the mean test scores for the experimental group (white circles) and the active control group (black circles). At baseline, there were no significant differences between groups (*p* = 1), indicating comparable starting points and minimizing the risk of baseline confounding. Over the intervention course, the experimental group exhibited a progressive and statistically significant improvement in test scores (I1: *p* < 0.001; I2: *p* < 0.001; I3: *p* = 0.013), whereas the active control group showed no improvement. These findings suggest that the in-phase bilateral upper limb exercise protocol led to a meaningful improvement in perceived quality of life in the experimental group.

Higher scores reflected better QoL. ANOVA (Tables S5–S8) and the individual results (Table S12) showed significant improvement in the experimental group from baseline (M = 88.8 (SD = 6.1)) to intervention (M = 95.6 (SD = 5.5); *p* = 0.029), with no change in the active control (baseline; M = 85.7 (SD = 8.3) to intervention; M = 85.6 (SD = 6.7)). A significant group-by-phase interaction was observed (*p* = 0.009) and the post hoc analysis confirmed within-group improvements in the experimental group only (Figure 4). An independent *t*-test further supported a greater improvement (*t*(18) = 7.03, *p* < 0.05) in the experimental group (M = 6.8 (SD = 2.3)) compared to the active control group (M = −0.06 (SD = 2.9)).

#### 3.2.2. Modified Fatigue Impact Scale

**Figure 5:**
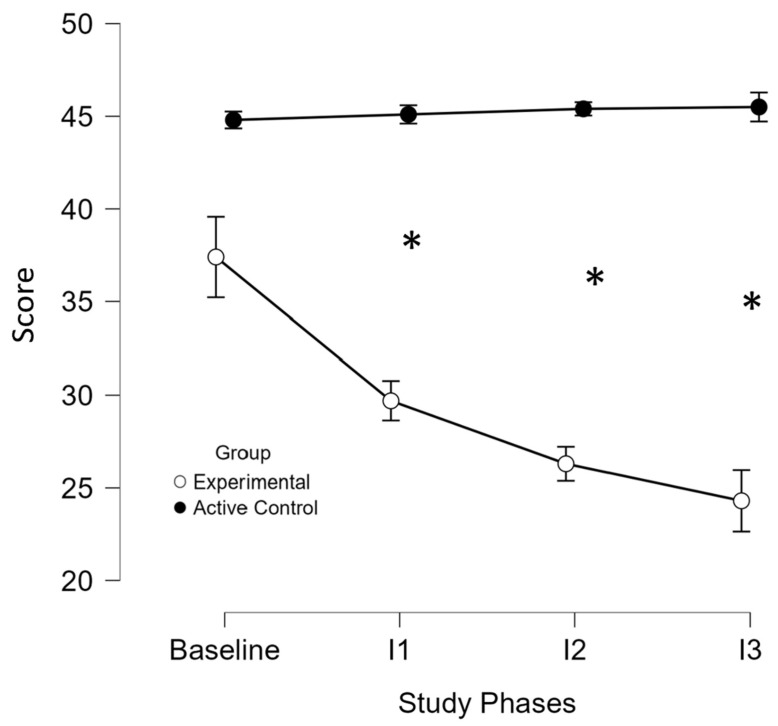
Performance of Modified Fatigue Impact Scale across study phases and groups. I, intervention; *, significant improvement (*p* ≤ 0.05) at the specified assessment point between the two study groups. The x-axis displays the baseline assessment (mean score) and three subsequent intervention time points (I1, I2, I3), while the y-axis represents the mean test scores for the experimental (white circles) and active control (black circles) groups. At baseline, the mean scores of the two groups did not significantly differ (*p* = 1), indicating comparable starting points and reducing the likelihood that initial differences influenced the observed outcomes. Over the course of the intervention, the experimental group exhibited a progressive and sustained improvement, reflected by a significant reduction in performance scores at each time point (I1: *p* = 0.004; I2: *p* < 0.001; I3: *p* < 0.001). In contrast, the active control group showed no significant change across the same period. These findings suggest that the in-phase bilateral upper limb exercise protocol led to a significant reduction in fatigue levels in the experimental group.

Lower scores indicated less fatigue. Fatigue significantly decreased in the experimental group only, with mean scores dropping from baseline M = 34 (SD = 14.8) to intervention M = 26.7 (SD = 15) (*p* < 0.001), while the active control group showed no change. ANOVA (Tables S19–S22) and the individual results (Table S3) confirmed a significant group-by-phases interaction (*p* < 0.001), with no baseline differences. Post hoc tests (Bonferroni-corrected) showed significant improvements at all intervention time points, in the experimental group only (Figure 5). A *t*-test confirmed greater improvement in the experimental group (M = 10.6 (SD = 7.9)) versus the active control group (M = −0.3 (SD = 1.6)), *t*(18) = 4.1, *p* < 0.05.

#### 3.2.3. Purdue Pegboard Test

**Figure 6:**
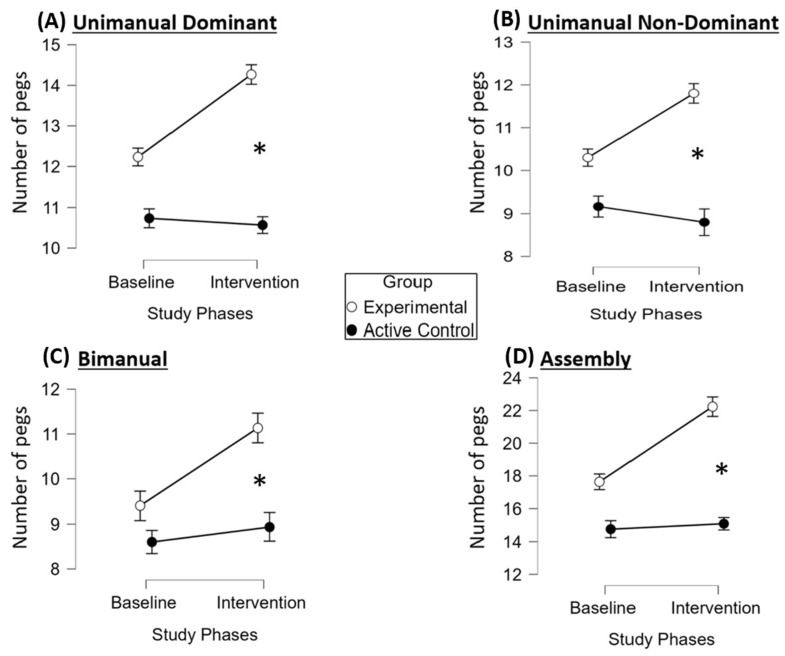
Performance on the Purdue Pegboard Test across study phases and groups. Abbreviation: *, significant improvement (*p* ≤ 0.05) at the specified assessment point between the two study groups. The mean assessment points per study phase are presented on the x-axis, whereas on the y-axis, the mean scores are shown for each Purdue Pegboard subtest, unimanual dominant (**A**), unimanual non-dominant (**B**), bimanual (**C**) and Assembly (**D**), at baseline and intervention. The experimental group (white circles) demonstrated significant improvements across all subtests (all *p* < 0.001), whereas this was not detected in the active control group (black circles), as indicated form the statistical analysis. Baseline characteristics of both groups, as indicated by their mean scores, showed no significant differences (all *p* = 1), suggesting that both groups started from comparable baselines, minimizing the potential influence of initial differences on the outcomes. These findings suggest enhanced manual dexterity following the in-phase bilateral upper limb exercise protocol.

Higher scores reflected improved performance on the PPT, indicating enhanced manual dexterity. Both experimental and active control groups showed changes from baseline to intervention across all subtests (Tables S4–S7). In the experimental group, mean scores increased in the unimanual dominant (baseline; M = 12.2 (SD = 2.3) to intervention; M = 14.2 (SD = 2.3)), unimanual non-dominant (baseline; M = 10.3 (SD = 2) to intervention; M = 11.8 (SD = 2.2)), bimanual (baseline; M = 9.4 (SD = 1.3) to intervention; M = 11.1 (SD = 1.7)) and Assembly (baseline; M = 17.6 (SD = 4.5) to intervention; M = 22.3 (SD = 5.4)) subtests, while the active control group exhibited minimal change (Figure 6).

ANOVA (Tables S23–S28) revealed significant effects of study phases and subtests (*p* < 0.001), with significant interactions (1) between study phases and group, (2) subtests and group (all *p* = 0.035), and (3) a three-way interaction among phases, subtests, and group (*p* < 0.001). Groups were equivalent at baseline (*p* = 1 for all subtests), confirming comparable starting points and reducing the likelihood that initial differences influenced outcomes. Bonferroni-corrected post hoc tests showed significant improvements in the experimental group for all subtests except unimanual non-dominant (*p* = 0.6), while no significant changes were observed in the active control group.

**Figure 7:**
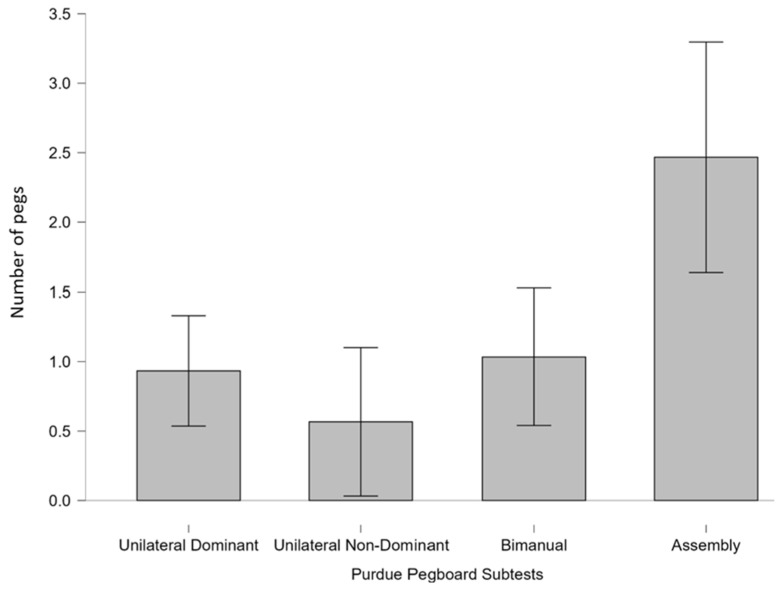
Mean improvement scores across Purdue Pegboard subtests for the experimental group. The Purdue Pegboard subtests are shown on the x-axis, while the y-axis represents improvement measured by the number of pegs for each subtest. Bar plot showing average improvement in performance (baseline—intervention) across the four subtests of the Purdue Pegboard Test: Unilateral Dominant, Unilateral Non-Dominant, bimanual, and Assembly. The largest improvement was observed in the Assembly subtest (4.6 pegs) compared to the other subtests, unimanual dominant (2 pegs), unimanual non-dominant (1.5 pegs), and bimanual (1.7 pegs), indicating greater enhancement in complex, coordinated manual dexterity following the in-phase bilateral upper limb exercise protocol.

The greatest improvement in the experimental group occurred in the Assembly subtest (+4.6 pegs), significantly exceeding improvements in other subtests (*p* < 0.01) (Figure 7). No significant within-group differences were found among other subtests or in the controls (*p* = 1). The results indicate that the in-phase bilateral upper limb exercise protocol led to greater improvements in manual dexterity compared to the conventional exercise program.

#### 3.2.4. Timed 25-Foot Walk

**Figure 8:**
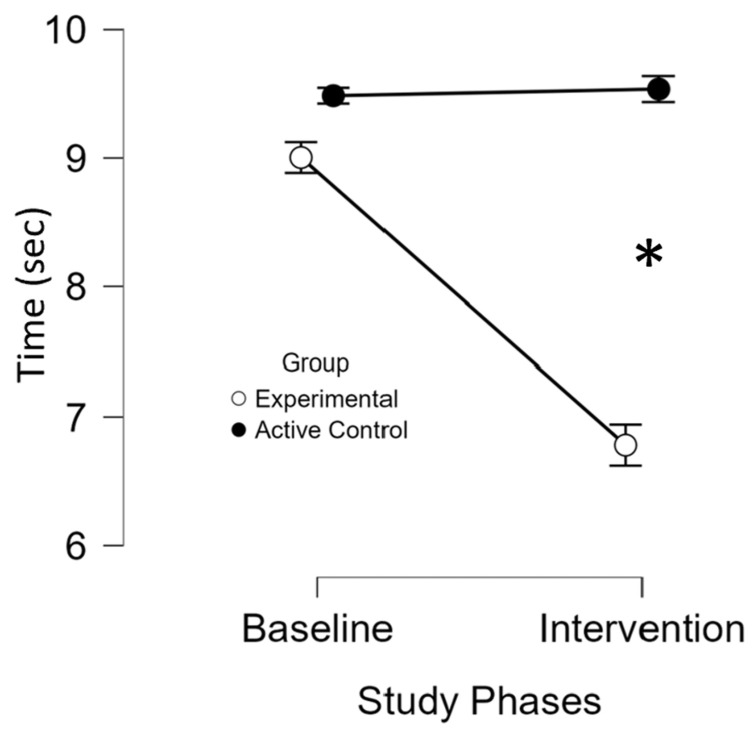
Performance on the Timed 25-Foot Walk Test across study phases and groups. *, significant improvement (*p* ≤ 0.05) at the specified assessment point between the two study groups. The x-axis represents the study phases, while the y-axis displays the mean test scores for the experimental (white circles) and active control (black circles) groups. Baseline comparisons between groups revealed no significant differences in mean scores (*p* = 1), indicating comparable starting points and reducing the likelihood that initial disparities influenced the outcomes. Over the intervention course, the experimental group exhibited a significant and sustained improvement (*p* < 0.001), reflected by a progressive decline in test scores. In contrast, the active control group showed no improvement. These findings suggest that the in-phase bilateral upper limb exercise protocol led to a significant enhancement in gait speed.

Lower values indicated better gait performance. In the experimental group, gait performance improved significantly with test mean time decreasing from baseline M = 9 (SD = 2.3) to intervention M = 6.7 (SD = 2) (*p* < 0.001), unlike controls (baseline; M = 4.4 (SD = 2.7) to intervention; M = 9.5 (SD = 2.8)). ANOVA (Tables S29–S32) and the individual results (Table S9) from both study groups showed a significant group-by-phase interaction (*p* < 0.001). Post hoc analysis and the *t*-tests confirmed greater improvement (*p* < 0.05) in the experimental group (M = 2.2 (SD = 0.7)) compared to the active control group (M = −0.05 (SD = 0.2)), with comparable baseline mean times (Figure 8).

#### 3.2.5. Six Spot Step Test

**Figure 9:**
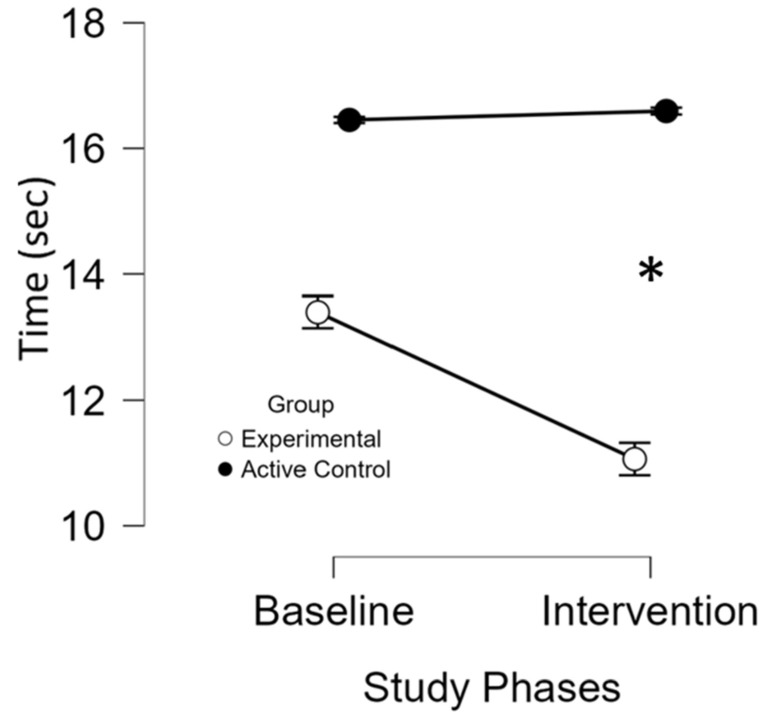
Performance on the Six Spot Step Test across study phases and groups. *, significant improvement (*p* ≤ 0.05) at the specified assessment point between the two study groups. The x-axis represents the study phases, while the y-axis displays the mean test scores for the experimental (white circles) and active control (black circles) groups. Baseline comparisons between groups revealed no significant differences in mean scores (*p* = 1), indicating comparable starting points and reducing the likelihood that initial disparities influenced the outcomes. Over the intervention course, the experimental group exhibited a significant and sustained improvement (*p* < 0.001), reflected by a progressive decline in test scores. In contrast, the active control group showed no improvement. These findings suggest that the in-phase bilateral upper limb exercise protocol led to a significant enhancement in gait speed.

Lower values indicate better lower limb function. Test completion time significantly improved in the experimental group (baseline; M = 13.3 (SD = 8.3) to intervention; M = 11 (SD = 6.6), *p* = 0.022) based on non-parametric Wilcoxon signed-rank test), with no change in controls (baseline; M = 16.4 (SD = 8.7) to intervention; M = 16.5 (SD = 8.9)) (Figure 9). Individual data is shown in Table S10.

### 3.3. Correlation Between SDMT and PPT

**Figure 10:**
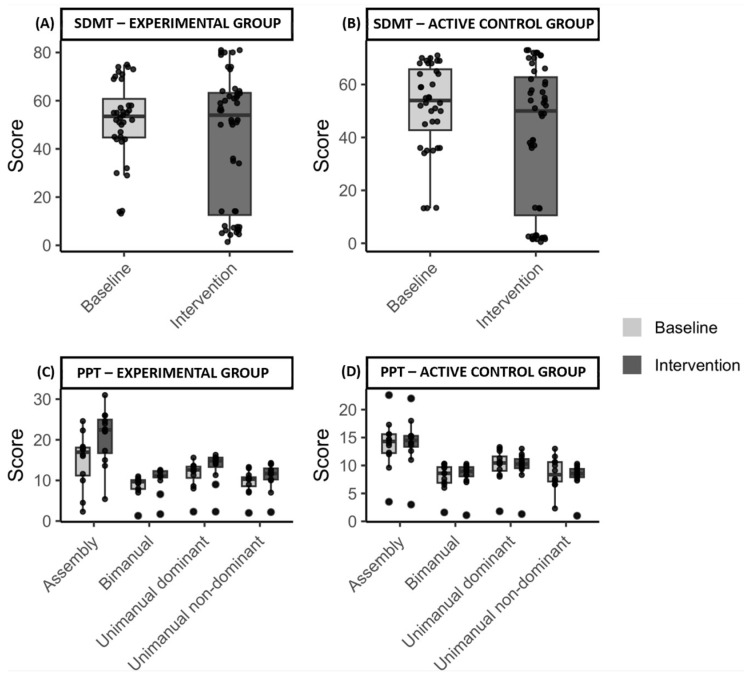
Individual and group results across Symbol Digit Modalities Test and Purdue Pegboard Test subtests. SDMT, Symbol Digit Modalities Test; PPT, Purdue Pegboard Test. Distribution of cognitive (i.e., SDMT) and manual dexterity test (i.e., PPT) scores across subtests for each group at baseline and intervention phases. Boxplots represent the median and interquartile range, while points show individual mean participant scores across the three baseline and intervention observations for (**A**) SDMT scores for the experimental group, (**B**) SDMT scores for the active control group, (**C**) PPT scores for the experimental group, and (**D**) PPT scores for the active control group.

The Pearson’s correlation coefficient (Pearson’s *r*) was calculated using the mean score from the intervention phase to examine the relationship between information processing speed and manual dexterity. The analysis included the SDMT and the subtests of the PPT. In the experimental group (Table 3), a non-significant moderate correlation was observed between the SDMT and the unimanual dominant hand dexterity (*r* = 0.6, *p* = 0.07), the unimanual non-dominant hand dexterity (*r* = 0.6, *p* = 0.09) and the bimanual dexterity (*r* = 0.5, *p* = 0.1). Individual results for each test are presented in Figure 10 and in the Supplementary Materials (Tables S1 and S4–S7). However, a significant moderate correlation was observed between the SDMT and the Assembly dexterity (*r* = 0.7, *p* = 0.02). On the other hand, in the active control group (Table 4), a non-significant weak correlation was found between the SDMT and the Purdue Pegboard subtests (unimanual dominant hand: *r* = 0.3, *p* = 0.4; unimanual non-dominant hand: *r* = 0.5, *p* = 0.1; bimanual: *r* = 0.5, *p* = 0.1; Assembly: *r* = 0.2, *p* = 0.6). Nevertheless, the highest significant correlation was found in the experimental group, between two couplings of Purdue Pegboard subtests, the unimanual non-dominant hand–bimanual (*r* = 0.9, *p* = 0.001) and the unimanual non-dominant hand–Assembly (*r* = 0.9, *p* = 0.001). Following Bonferroni correction for the five tests, a statistically significant *p*-value is equal or less than 0.01 (*p* = 0.05/number of tests = 5).

## 4. Discussion

Exercise has increasingly been recognized as a comprehensive rehabilitation strategy capable of concurrently addressing both cognitive and motor domains [3,4], thereby offering broader therapeutic benefits than cognitive training alone [6]. This pilot study sought to investigate the potential efficacy of in-phase bilateral upper limb exercises in pwPMS, yielding several novel findings. First, participation in a 12-week exercise protocol, based on in-phase bilateral upper limb movements, led to significant improvements in information processing speed; however, these cognitive gains did not correlate significantly with changes in manual dexterity. Second, the specific exercise protocol elicited marked improvements in balance, gait, fatigue, and QoL when compared with the conventional exercise program.

In-phase Bilateral Exercises—Information Processing Speed

Statistical analysis of the SDMT revealed significant improvements in both groups, indicating enhanced information processing speed in pwPMS [53,54], with significantly greater gains observed in the experimental group. As no participants engaged in structured exercise prior to the intervention, baseline cognitive function remained stable, suggesting that observed improvements were attributable to the intervention.

Information processing speed is a critical prognostic indicator of physical impairment [55] and represents the most prevalent cognitive deficit [3,4,56] in pwPMS [53,54]. These findings align with prior research demonstrating the cognitive benefits of exercise in this population [57,58,59,60,61], including our previous study showing enhanced cognitive processing performance following in-phase bilateral upper limb exercises in individuals with Relapsing-Remitting MS [62].

Emerging evidence supports a strong relationship between motor function and cognition in both healthy and MS populations [11,12,63,64,65]. The anterior cingulate cortex, particularly its dorsal division, plays a central role in executive functions (i.e., attention, cognitive processing) [66,67] and is also activated during motor tasks [13,16,68]. This region is a key node in motor–cognitive integration due to its dense projections to the motor cortex and spinal cord [13,15,16]. Asemi et al. (2015) demonstrated that the dorsal anterior cingulate cortex modulates activity in the supplementary motor area, reinforcing its role in motor control [13]. Additionally, bimanual coordination has been linked to enhanced intrahemispheric and transcallosal connectivity between the supplementary motor area and primary motor cortex [13,17,18,69]. Grefkes et al. (2008) reported that in-phase bilateral upper limb movements improve interhemispheric communication, supporting the cognitive–motor integration hypothesis [18]. Based on our findings and the previous mentioned evidence, the in-phase bilateral upper limb exercise protocol enhances information processing speed in pwPMS, supporting its integration into neurorehabilitation.

In-phase Bilateral Exercises—Manual dexterity

Our findings showed greater improvement in manual dexterity in the experimental group. Despite higher baseline scores, post hoc analysis confirmed no significance between-group differences, indicating comparable initial performance across subtests.

Following the in-phase bilateral exercise protocol, the experimental group exhibited significantly greater improvements across all subtests, with the most notable gains in the Assembly task, the most cognitively demanding measure of manual dexterity [70,71]. In contrast, controls showed no significant changes. While improvements in the unimanual non-dominant subtest did not reach statistical significance, the observed trend aligns with prior research supporting the transfer effects of bilateral training [72,73] and its role in reducing lateral asymmetries [74].

These improvements may involve corpus callosum-mediated mechanisms that enhance bimanual coordination [75] and facilitate unilateral motor performance [76]. Studies by Seitz et al. (2004) and Smith & Staines (2010) demonstrated that in-phase bimanual training increases cortical activation and improves motor performance in both clinical and healthy populations [76,77]. Given that lesions in the corpus callosum are common in people with MS and can impair bimanual coordination [78,79], our findings, consistent with those of Seitz and Smith & Staines, suggest that in-phase bilateral upper limb exercises may enhance bimanual dexterity in pwPMS.

Previous studies have shown that task-specific and intensive training is crucial for inducing neuroplastic changes in the non-dominant limb. The 12-week in-phase bilateral exercise protocol with three sessions per week may have been insufficient, as longer and more frequent interventions are often required to produce measurable changes [80,81,82]. Nonetheless, the observed bilateral benefits indicate that this approach may be particularly valuable for individuals with motor impairments, who frequently underuse the non-dominant or affected upper limb.

Information Processing Speed—Manual Dexterity Correlation

A Pearson’s correlation analysis was performed between SDMT and PPT to define if the improvement in information processing speed influences manual dexterity. Therefore, a moderate correlation emerged, with statistical significance observed only between SDMT and the Assembly subtest (Table 3 and Table 4). This linear relationship was present in the experimental group but absent in the active control group. These results are consistent with prior findings in pwPMS [12,64,69], individuals with mild cognitive impairment and healthy older adults [69,83]. Notably, the Assembly subtest, the most cognitively demanding task in the PPT, showed the greatest improvement among experimental participants. The Assembly task requires advanced cognitive control [70,71], including sequential planning and coordinated use of both hands within time constraints. This supports that in-phase bilateral upper limb exercises may have the potential to simultaneously engage cognitive processing and manual dexterity. Such behavioral associations were not observed in the active control group, which engaged in conventional exercises. However, given the small sample size (n = 10 per group), these results should be interpreted as preliminary and hypothesis-generating rather than conclusive.

In-phase Bilateral Exercises—Secondary Outcome Measures

Participants in the experimental group showed significant improvements across all exploratory secondary outcomes compared with controls. Although the exercise protocol in the experimental group primarily targeted upper limb function, its circuit-based design likely contributed to the observed enhancements in gait and balance [84,85], as it incorporated both gait and balance practice through transitions between exercises. Furthermore, significant reductions in fatigue and improvements in QoL were observed, as measured by the Modified Fatigue Impact Scale and the Medical Outcomes Study Short Form-36 Health Survey. These findings align with previous research linking exercise to improved cognition, manual dexterity, and QoL in pwPMS [86,87,88], despite the commonly reported increase in fatigue perception that often accompanies neurological improvement in this population [89,90,91,92].

The experimental group showed a significant improvement in cognitive processing speed alongside a marked reduction in fatigue, whereas the active control group demonstrated no meaningful changes despite comparable baseline cognitive performance. This parallel improvement supports the notion that fatigue may modulate cognitive processing speed in pwPMS. Fatigue has been consistently associated with impaired information processing speed in MS, particularly in progressive phenotypes [91,92]. The absence of cognitive improvement in the control group further suggests that the observed gains were unlikely to be attributable to practice effects alone and may be partly mediated by reductions in fatigue. Although examining the correlation between changes in fatigue and information processing speed was beyond the scope of the present study, future research should formally investigate this relationship in pwPMS.

Taken together, the observed improvements across cognitive and motor domains highlight the potential of in-phase bilateral upper limb exercises as a comprehensive rehabilitation strategy for pwPMS. These findings can be interpreted within a multidimensional, data-informed decision-making framework, which provides a structured approach for integrating heterogeneous outcome measures and guiding individualized rehabilitation planning. Such a framework has been applied in other domains, such as in sports performance optimization, where cognitive, motor, and functional data are combined to inform evidence-based interventions [93]. Adapting this conceptual approach to neurorehabilitation may facilitate the translation of multimodal patient data into actionable, patient-specific strategies, supporting more precise and holistic treatment planning.

### 4.1. Limitations

The present study highlights several methodological considerations that should be addressed in future research. Firstly, concerning the use of the MMSE as an exclusion criterion for cognitive screening, while effective for detecting overt impairment, it is less sensitive than the Montreal Cognitive Assessment and MS-specific brief batteries (e.g., BICAMS, MACFIMS, Rao’s Brief Repeatable Battery) for detecting subtle deficits, particularly in younger participants. At the time, the MMSE was the standard tool in our clinical setting, ensuring feasibility and consistency. Future studies shoulder more sensitive instruments to better screen cognitive performance in people with MS. Secondly, the absence of a follow-up assessment limits the ability to evaluate the durability and reliability of the observed effects, as well as to capture potential long-term benefits for participants. Thirdly, the relatively small sample size, although appropriate for a pilot study aimed at generating preliminary data to inform future trials, may constrain the generalizability of the findings.

Moreover, we acknowledge that the unequal frequency of exercise sessions between groups may have influenced the interpretation of outcomes, highlighting the importance of using standardized training protocols in future studies to improve comparability. However, as a pilot randomized controlled trial, the present study was primarily designed to evaluate the feasibility and preliminary efficacy of the in-phase bilateral intervention, rather than to isolate the specific effects of training frequency. Future larger-scale trials with standardized intervention dosages should address this aspect to determine the optimal exercise parameters for maximizing benefits. Consequently, the current study design does not permit differentiation of the effects attributable to exercise frequency and the findings should therefore be interpreted within an exploratory framework. Nevertheless, this study contributes to addressing a critical gap highlighted by De Luca et al. (2020) concerning the optimization of intervention dosage in people with MS [4].

Similarly, we acknowledge that, although the experimental group participated in a group-based exercise program, exercise rehabilitation is increasingly shifting toward individually prescribed protocols. Within this conceptual framework, group-based exercise in the present study was not implemented as an experimental variable, but rather as a strategy to enhance motivation, adherence, and social engagement, factors known to positively influence cognitive outcomes. The differential effects of group-based versus individually delivered interventions, however, were beyond the scope of the current investigation.

Finally, the absence of neuroimaging or other qualitative outcome measures, such as functional magnetic resonance imaging, limits the ability to infer the neural mechanisms underlying the observed cognitive and motor improvements.

### 4.2. Future Directions

To build upon the present findings, future research should aim to replicate the present in-phase bilateral upper limb exercise protocol in larger cohorts of pwPMS to improve statistical power and generalizability. The use of more sensitive and MS-specific cognitive screening is recommended to better detect subtle cognitive performance. Longitudinal designs incorporating follow-up assessments are needed to evaluate the durability and clinical relevance of exercise-induced effects. In addition, future trials should standardize intervention dosage across study arms to disentangle the effects of training frequency from intervention type and to identify optimal exercise parameters. The inclusion of quantitative neuroimaging techniques, such as functional magnetic resonance imaging, would facilitate the investigation of the neural mechanisms underlying observed cognitive and motor improvements. Finally, although group-based exercise was employed in this pilot study to enhance motivation and adherence, future studies should directly compare group-based and individually prescribed delivery formats to clarify their relative contributions to neurorehabilitation outcomes.

## 5. Conclusions

Over recent decades, exercise has become a cornerstone in managing cognitive and motor impairments in pwPMS. Consistent with De Luca et al. (2020), who emphasized the need for holistic interventions targeting both domains [4], this pilot study examined the effects of in-phase bilateral upper limb exercises on information processing speed in pwPMS. The group-based, circuit-format protocol was designed to concurrently engage cognitive and motor functions.

Preliminary findings suggest that in-phase bilateral training is associated with improvements in cognitive processing and bimanual coordination, functions that rely on higher-order cognitive control [24,25,26]. Given that in-phase bilateral exercises impose lower attentional and motor demands than unilateral exercises [24,25,26], the observed trends in information processing speed and manual dexterity provide preliminary evidence supporting the feasibility of this approach as a targeted rehabilitation strategy in pwPMS. Although this pilot study had a small sample size and some variability in intervention dose, it suggests that a holistic, circuit-based, in-phase bilateral exercise program, combining sports activities and functional exercises, may provide preliminary benefits for both motor and cognitive functions in pwPMS. These findings warrant further investigation in larger mechanistic trials to clarify potential effects and underlying neurophysiological mechanisms.

## Figures and Tables

**Table 1:** Participants’ demographic and baseline clinical characteristics.

Participant	Age (Years)	Gender	Dominant Hand	Disease Duration	EDSS	MMSE
Experimental Group						
1	47	Female	Right	12	3	30
2	45	Male	Right	18	3	30
3	56	Female	Right	10	3.5	29
4	59	Male	Right	13	3.5	28
5	49	Female	Right	15	3.5	30
6	66	Male	Right	9	4	26
7	60	Male	Right	23	4	28
8	59	Female	Right	17	4	30
9	59	Female	Left	11	5	26
10	58	Female	Right	22	5	27
Mean (SD)	55.8 (6.6)	Females (total n = 6)	Left (total n = 1)	15 (4.9)	3.8 (0.7)	28.4 (1.6)
Active Control Group						
1	61	Female	Right	20	3	29
2	52	Male	Right	11	3.5	28
3	63	Male	Left	11	3.5	29
4	48	Male	Right	12	3.5	30
5	56	Female	Right	9	4	30
6	63	Female	Right	16	4	30
7	51	Female	Right	14	4	28
8	44	Male	Right	13	4.5	26
9	65	Male	Right	12	4.5	30
10	56	Female	Right	10	5	29
Mean (SD)	55.9 (7)	Females (total n = 5)	Left (total n = 1)	2.8 (3.2)	3.9 (0.6)	28.9 (1.2)

EDSS, Expanded Disability Status Scale; MMSE, Mini Mental State Examination; SD, Standard Deviation. The mean EDSS score was 3.8 in the experimental group and 3.9 in the active control group, both indicating moderate to significant disability. The gender distribution in the experimental group was skewed towards females (female-to-male ratio 6:4), whereas the active control group had an equal distribution (5:5). Handedness was comparable between groups, with nine out of ten participants in each group being right-handed. The mean age was 55.8 years in the experimental group and 55.9 years in the active control group. Cognitive function, as assessed by the MMSE, was preserved in both groups, with mean scores of 28.4 in the experimental and 28.9 in the active control groups, indicating no evidence of cognitive impairment.

**Table 2:** Overview of the exercise protocol.

Exercise Number	Type of Exercise	Duration	Body Position	Difficulty Level
1	Basketball chest pass	1 min	Standing	Distance of the pass
2	PNF 1st diagonal FP	1 min	Standing	Elastic band
3	Shoulder flexion/extension	1 min	Sitting	Hand dumbbells
4	Adductor squeeze	1 min	Supine lying	Pilates ring
5	Basketball shoulder pass	1 min	Standing	Distance of the pass
6	PNF 1st diagonal EP	1 min	Standing	Elastic band
7	Shoulder abduction/adduction	1 min	Sitting	Hand dumbbells
8	Hip abduction	1 min	Supine lying	Pilates ring
9	Volleyball overhead pass	1 min	Standing	Distance of the pass
10	PNF 2nd diagonal FP	1 min	Standing	Elastic band
11	PNF 2nd diagonal EP	1 min	Standing	Elastic band
12	Squat	1 min	Standing	Balance pads

PNF, Proprioceptive Neuromuscular Facilitation; FP, Flexion Pattern; EP, Extension Pattern; min, Minute. Each session included three sets of nine different exercises which targeted large muscle groups of the upper limbs (i.e., 1–3, 5–7, 9–11) and three exercises which targeted large muscle groups of the lower limbs (i.e., 4, 8, 12). Overall, for all participants, the duration for each exercise was one minute; however, they were instructed to stop if they felt tired. The difficulty level of the sport activity tasks (i.e., 1, 5, 9) was maintained by changing the distance of the passes from the wall. The difficulty level of upper limb exercises 2, 6, 10, and 11 was adjusted by changing the resistance of elastic bands, while the difficulty of exercises 3 and 7 was modified by varying the weight of hand dumbbells.

**Table 3:** Correlations between improvements in Purdue Pegboard subtests and Symbol Digit Modalities Test for the experimental group.

Variables	SDMT		Dominant Hand		Non- Dominant Hand		Bimanual		Assembly	
	*r*	*p*- Value	*r*	*p*- Value	*r*	*p*- Value	*r*	*p*- Value	*r*	*p*- Value
SDMT	-	-	-	-	-	-	-	-	-	-
Dominant Hand	0.6	0.07	-	-	-	-	-	-	-	-
Non- Dominant Hand	0.6	0.09	0.8	0.006 *	-	-	-	-	-	-
Bimanual	0.5	0.1	0.8	0.01 *	0.9	0.001 *	-	-	-	-
Assembly	0.7	0.02	0.8	0.007 *	0.9	0.001 *	0.8	0.006 *	-	-

SDMT, Symbol Digit Modalities Test; *r*, Pearson correlation coefficient; *, significant after Bonferroni correction at *p* ≤ 0.05. In the experimental group, non-significant moderate correlation was observed between the SDMT and the unimanual dominant (*r* = 0.6, *p* = 0.07), the unimanual non-dominant (*r* = 0.6, *p* = 0.09) and the bimanual (*r* = 0.5, *p* = 0.1) subtests. However, significant moderate correlation was observed between the SDMT and the Assembly subtest (*r* = 0.7, *p* = 0.02). The highest correlation was observed between the unimanual dominant and bimanual subtests (*r* = 0.9, *p* = 0.001) and the unimanual dominant and Assembly subtests (*r* = 0.9, *p* = 0.001).

**Table 4:** Correlations between improvements in Purdue Pegboard subtests and Symbol Digit Modalities Test for the active control group.

Variables	SDMT		Dominant Hand		Non- Dominant Hand		Bimanual		Assembly	
	*r*	*p*- Value	*r*	*p*- Value	*r*	*p*- Value	*r*	*p*- Value	*r*	*p*- Value
SDMT	-	-	-	-	-	-	-	-	-	-
Dominant Hand	0.3	0.4	-	-	-	-	-	-	-	-
Non- Dominant Hand	0.5	0.1	0.7	0.02	-	-	-	-	-	-
Bimanual	0.5	0.1	0.6	0.05	0.5	0.1	-	-	-	-
Assembly	0.2	0.6	0.7	0.04	0.3	0.3	0.2	0.5	-	-

SDMT, Symbol Digit Modalities Test; *r*, Pearson correlation coefficient. In the active control group, non-significant weak correlation was found between the SDMT and the PPT (unimanual dominant hand: *r* = 0.3, *p* = 0.4; unimanual non-dominant hand: *r* = 0.5, *p* = 0.1; bimanual: *r* = 0.5, *p* = 0.1; Assembly: *r* = 0.2, *p* = 0.6).

## Data Availability

Data are available at FIGSHARE: https://doi.org/10.6084/m9.figshare.26953714.v1.

## Supplementary Materials

The following supporting information can be downloaded at: https://www.mdpi.com/article/10.3390/brainsci16020191/s1. Table S1. Symbol Digit Modalities Test group’s data across all assessment points during baseline and intervention phases. Table S2. Medical Outcomes Study Questionnaire Short Form 36 Health Survey group’s data across all assessment points during baseline and intervention phases. Table S3. Modified Fatigue Impact Scale group’s data across all assessment points during baseline and intervention phases. Table S4. Purdue Pegboard Test—unimanual dominant hand subtest—group’s data across all assessment points during baseline and intervention phases. Table S5. Purdue Pegboard Test—unimanual non-dominant hand subtest—group’s data across all assessment points during baseline and intervention phases. Table S6. Purdue Pegboard Test—bimanual subtest—group’s data across all assessment points during baseline and intervention phases. Table S7. Purdue Pegboard Test—Assembly subtest—group’s data across all assessment points during baseline and intervention phases. Table S8. Purdue Pegboard Subtests—improvement between groups. Table S9. Timed 25-Foot Walk Test group’s data across all assessment points during basely and intervention phases. Table S10. Six Spot Step Test group’s data across all assessment points during baseline and intervention phases. Table S11. Symbol Digit Modalities Test—within-subject effects. Table S12. Symbol Digit Modalities Test—post hoc comparisons—study phases. Table S13. Symbol Digit Modalities Test—post hoc comparisons—group ✻ study phases. Table S14. Symbol Digit Modalities Test—*t*-test—improvement differences between groups. Table S15. Medical Outcomes Study Questionnaire Short Form 36 Health Survey—within-subject effects. Table S16. Medical Outcomes Study Questionnaire Short Form 36 Health Survey—post hoc comparisons—study phases. Table S17. Medical Outcomes Study Questionnaire Short Form 36 Health Survey—post hoc comparisons—group ✻ study phases. Table S18. Medical Outcomes Study Questionnaire Short Form 36 Health Survey—*t*-test—improvement differences between groups. Table S19. Modified Fatigue Impact Scale—within-subject effects. Table S20. Modified Fatigue Impact Scale—post hoc comparisons—study phases. Table S21. Modified Fatigue Impact Scale—post hoc comparisons—group ✻ study phases. Table S22. Modified Fatigue Impact Scale—*t*-test—improvement differences between groups. Table S23. Purdue Pegboard Test—within-subject effects. Table S24. Purdue Pegboard test—post hoc comparisons—study phases. Table S25. Purdue Pegboard test—post hoc comparisons—subtests. Table S26. Purdue Pegboard test—post hoc comparisons—group ✻ study phases ✻ subtests. Table S27. Purdue Pegboard Subtests improvement—within-subject effects. Table S28. Purdue Pegboard subtest improvement—post hoc comparisons—group ✻ subtests. Table S29. Timed 25-Foot Walk Test—within-subject effects. Table S30. Timed 25-Foot Walk Test—post hoc comparisons—study phases. Table S31. Timed 25-Foot Walk Test—post hoc comparisons—group ✻ study phases. Table S32. Timed 25-Foot Walk Test—*t*-test—improvement differences between groups. Table S33. Six Spot Step Test—within-subject effects. Table S34. Six Spot Step Test—post hoc comparisons—study—study phases. Table S35. Six Spot Step Test—post hoc comparisons—group ✻ study phases. Table S36. Six Spot Step Test—*t*-test—improvement differences between groups.

## Abbreviations

The following abbreviations are used in this manuscript:

MS	Multiple Sclerosis
PMS	Progressive Multiple Sclerosis
pwPMS	People with Progressive Multiple Sclerosis
QoL	Quality of Life
EDSS	Expanded Disability Status Scale
MMSE	Mini Mental State Examination
SDMT	Symbol Digit Modalities Test
PPT	Purdue Pegboard Test
M	Mean
SD	Standard Deviation
