# Supplementary material for: In-Phase Bilateral Upper Limb Exercises Improve Cognitive and Motor Functions in Progressive Multiple Sclerosis: A Pilot Randomized Controlled Trial
Source: Brain Sci. 2026 Feb 5;16(2):191. doi: 10.3390/brainsci16020191 (PMC12938636; doi:10.3390/brainsci16020191)
Supplement: Supplementary file 1 [file brainsci-16-00191-s001.zip › brainsci-4133005-supplementary.pdf]

brain sciences

MDPI

Section S1_Individual results based on the outcome measure and group allocation.

Table S1. Symbol Digit Modalities Test groups data across all assessment points during baseline and intervention phases.

Experimental Group									
Participant	B1	B2	B3	Mean	I1	I2	I3	Mean	Improve
1	47 (0)	45 (0)	45 (0)	45.6 (0)	50 (0)	52 (0)	51 (0)	51 (0)	5.3 (0)
2	51 (0)	51 (0)	52 (0)	51.3 (0)	57 (0)	56 (0)	56 (0)	56.3 (0)	5 (0)
3	55 (0)	54 (0)	56 (0)	55 (0)	62 (0)	64 (0)	63 (0)	63 (0)	8 (0)
4	44 (0)	44 (0)	43 (0)	43.6 (0)	50 (0)	51 (0)	52 (0)	51 (0)	7.3 (0)
5	69 (0)	70 (0)	69 (0)	69.3 (0)	74 (0)	73 (0)	74 (0)	73.6 (0)	4.3 (0)
6	74 (0)	73 (0)	74 (0)	73.6 (0)	79 (0)	81 (0)	80 (0)	80 (0)	6.3 (0)
7	72 (0)	75 (0)	71 (0)	72.6 (0)	80 (0)	81 (0)	80 (0)	80.3 (0)	7.6 (0)
8	57 (0)	58 (0)	58 (0)	57.6 (0)	63 (0)	65 (0)	64 (0)	64 (0)	6.3 (0)
9	52 (0)	50 (0)	53 (0)	51.6 (0)	59 (0)	59 (0)	60 (0)	59.3 (0)	7.6 (0)
10	32 (0)	30 (0)	29 (0)	30.3 (0)	35 (0)	36 (0)	34 (0)	35 (0)	4.6 (0)
**Mean**	**55.3 (0)**	**55 (0)**	**55 (0)**	**55.1 (0)**	**60.9 (0)**	**61.8 (0)**	**61.4 (0)**	**61.3 (0)**	**6.2 (0)**
**SD**	**13.2 (0)**	**14.3 (0)**	**13.9 (0)**	**13.8 (0)**	**14.1 (0)**	**14.1 (0)**	**14.2 (0)**	**14.1 (0)**	**1.4 (0)**
Active Control Group									
1	50 (0)	51 (0)	50 (0)	50.3 (0)	53 (0)	51 (0)	52 (0)	52 (0)	1.6 (0)
2	36	35	34	35	38	36	37	37	2

	(0)	(0)	(0)	(0)	(0)	(0)	(0)	(0)	(0)
3	35	36	36	35.6	37	39	39	38.3	2.6
	(0)	(0)	(0)	(0)	(0)	(0)	(0)	(0)	(0)
4	59	60	59	59.3	61	60	62	61	1.6
	(0)	(0)	(0)	(0)	(0)	(0)	(0)	(0)	(0)
5	64	64	65	64.3	65	66	68	66.3	2
	(0)	(0)	(0)	(0)	(0)	(0)	(0)	(0)	(0)
6	70	71	70	70.3	72	73	71	72	1.6
	(0)	(0)	(0)	(0)	(0)	(0)	(0)	(0)	(0)
7	69	69	68	68.6	70	71	73	71.3	2.6
	(0)	(0)	(0)	(0)	(0)	(0)	(0)	(0)	(0)
8	45	46	46	45.6	49	48	49	48.6	3
	(0)	(0)	(0)	(0)	(0)	(0)	(0)	(0)	(0)
9	53	52	53	52.6	54	54	55	54.3	1.6
	(0)	(0)	(0)	(0)	(0)	(0)	(0)	(0)	(0)
10	68	68	69	68.3	72	70	72	71.3	3
	(0)	(0)	(0)	(0)	(0)	(0)	(0)	(0)	(0)
**Mean**	**54.9**	**55.2**	**55**	**55**	**57.1**	**56.8**	**57.8**	**57.2**	**2.2**
	**(0)**	**(0)**	**(0)**	**(0)**	**(0)**	**(0)**	**(0)**	**(0)**	**(0)**
**SD**	**13.2**	**13.3**	**13.4**	**13.3**	**13.1**	**13.3**	**13.4**	**13.2**	**0.5**
	**(0)**	**(0)**	**(0)**	**(0)**	**(0)**	**(0)**	**(0)**	**(0)**	**(0)**

B, Baseline; I, Intervention; SD, Standard Deviation. Values were expressed in numbers, numbers in briefcases refer to the mistakes which they were done in each assessment point. Improvement was calculated as the difference between B and mean I (i.e., Improve = mean Intervention – mean Baseline).

Table S2. Medical Outcomes Study Questionnaire Short Form 36 Health Survey groups data across all assessment points during baseline and intervention phases.

Experimental Group						
**Participant**	**B**	**I1**	**I2**	**I3**	**Mean I**	**Improve**
1	81	83	87	88	86	5
2	82	87	86	90	87.6	5.6
3	85	92	94	94	93.3	8.3
4	91	98	99	101	99.3	8.3
5	85	90	92	97	93	8
6	100	103	104	105	104	4

7	93	99	104	105	102.6	9.6
8	94	99	97	99	98.3	4.3
9	85	94	95	97	95.3	10.3
10	92	94	95	100	96.3	4.3
**Mean**	**88.8**	**93.9**	**95.3**	**97.6**	**95.6**	**6.8**
**SD**	**6.1**	**5.8**	**5.7**	**5.4**	**5.5**	**2.3**
			**Active Control Group**			
1	87	86	90	93	89.6	2.6
2	88	87	83	86	85.3	-2.6
3	81	81	81	80	80.6	-0.3
4	79	80	78	77	78.3	-0.6
5	81	81	83	82	82	1
6	101	103	103	76	94	-7
7	72	73	72	77	74	2
8	87	88	88	87	87.6	0.6
9	96	98	97	96	97	1
10	85	85	88	90	87.6	2.6
**Mean**	**85.7**	**86.2**	**86.3**	**84.4**	**85.6**	**0**
**SD**	**8.3**	**8.3**	**8.5**	**6.7**	**6.7**	**2.9**

B, Baseline; I, Intervention; SD, Standard Deviation. Values were expressed in numbers. Improvement was calculated as the difference between B and mean I Improvement was calculated as the difference between B and mean I (i.e., Improve = mean Intervention – mean Baseline). All negative values indicate no individual improvement.

Table S3. Modified Fatigue Impact Scale groups data across all assessment points during baseline and intervention phases.

			**Experimental Group**			
**Participant**	**B**	**I1**	**I2**	**I3**	**Mean I**	**Improve**
1	34	30	30	31	30.3	3.6
2	52	49	48	48	48.3	3.6
3	28	21	15	16	17.3	10.6
4	14	12	10	4	8.6	5.3
5	53	46	43	43	44	9
6	43	18	15	15	16	27

7	34	26	15	3	14.6	19.3
8	16	15	14	11	13.3	2.6
9	44	30	27	27	28	16
10	56	50	46	45	47	9
**Mean**	**34**	**29.7**	**26.3**	**24.3**	**26.7**	**10.6**
**SD**	**14.8**	**14.1**	**14.7**	**16.9**	**15**	**7.9**
**Active Control Group**						
1	49	50	52	52	51.3	-2.3
2	48	48	49	50	49	-1
3	81	82	80	81	81	0
4	23	24	22	24	23.3	-0.3
5	66	65	66	66	65.6	0.3
6	48	47	48	49	48	0
7	35	34	35	29	32.6	2.3
8	26	29	27	26	27.3	-1.3
9	17	17	16	16	16.3	0.6
10	55	55	59	62	58.6	-3.6
Mean	**44.8**	**50**	**52**	**52**	**45.3**	**-0.5**
SD	**19.9**	**19.7**	**20.3**	**21.1**	**20.3**	**1.6**

B, Baseline; I, Intervention; SD, Standard Deviation. Values were expressed in numbers. Improvement was calculated as the difference between B and mean I Improvement was calculated as the difference between B and mean I (i.e., Improve = mean Intervention – mean Baseline). All negative values indicate no individual improvement.

Table S4. Purdue Pegboard Test - Unimanual dominant hand subtest, groups data across all assessment points during baseline and intervention phases.

**Experimental Group**									
**Participant**	**B1**	**B2**	**B3**	**Mean**	**I1**	**I2**	**I3**	**Mean**	**Improve**
1	8	8	8	8	9	9	9	9	1
2	14	13	14	13.6	15	16	16	15.6	2
3	11	12	11	11.3	15	16	16	15.6	4.3
4	13	13	12	12.6	15	15	15	15	2.4
5	14	13	14	13.6	15	16	16	15.6	2
6	16	16	15	15.6	16	16	17	16.3	0.7

7	13	12	13	12.6	14	14	14	14	1.4
8	13	13	12	12.6	15	16	15	15.3	2.7
9	13	14	13	13.3	15	14	15	14.6	1.4
10	9	8	9	8.6	10	12	12	11.3	2.7
**Mean**	**12.4**	**12.2**	**12.1**	**12.2**	**13.9**	**14.4**	**14.5**	**14.2**	**2**
**SD**	**2.4**	**2.4**	**2.2**	**2.3**	**2.3**	**2.3**	**2.3**	**2.3**	**1**
**Active Control Group**									
1	10	10	11	10.3	9	10	9	9.3	-1
2	8	8	8	8	8	9	8	8.3	0.3
3	11	11	9	10.3	12	10	11	11	0.7
4	8	8	9	8.3	9	10	10	9.6	1.3
5	13	12	14	13	11	12	12	11.6	-1.4
6	12	10	10	10.6	12	10	10	10.6	0
7	13	13	14	13.3	12	12	12	12	-1.7
8	14	13	11	12.6	13	13	13	13	0.4
9	8	8	12	9.3	9	10	11	10	0.7
10	11	12	11	11.3	10	9	11	10	-1.3
Mean	**10.8**	**10.5**	**10.9**	**10.7**	**10.5**	**10.5**	**10.7**	**10.5**	**-0.2**
SD	**2.2**	**2**	**2**	**1.8**	**1.7**	**1.3**	**1.4**	**1.3**	**0.9**

B, Baseline; I, Intervention; SD, Standard Deviation. Values were expressed in numbers. Improvement was calculated as the difference between B and mean I Improvement was calculated as the difference between B and mean I (i.e., Improve = mean Intervention – mean Baseline). All negative values indicate no individual improvement.

Table S5. Purdue Pegboard Test - Unimanual non-dominant hand subtest, groups data across all assessment points during baseline and intervention phases.

**Experimental Group**									
**Participant**	**B1**	**B2**	**B3**	**Mean**	**I1**	**I2**	**I3**	**Mean**	**Improve**
1	8	7	6	7	7	7	7	7	0
2	9	9	9	9	9	10	11	10	1
3	11	11	10	10.6	12	12	11	11.6	1
4	11	10	10	10.3	15	13	14	14	3.7
5	13	13	13	13	15	14	14	14.3	1.3
6	13	14	13	13.3	14	14	14	14	0.7

7	11	10	11	10.6	12	13	12	12.3	1.7
8	12	11	11	11.3	12	12	14	12.6	1.3
9	10	10	11	10.3	11	12	12	11.6	1.3
10	7	7	8	7.3	10	10	11	10.3	3
**Mean**	**10.5**	**10.2**	**10.2**	**10.3**	**11.7**	**11.7**	**12**	**11.8**	**1.5**
**SD**	**2**	**2.2**	**2.1**	**2**	**2.5**	**2.1**	**2.2**	**2.2**	**1**
**Active Control Group**									
1	7	8	8	7.6	9	7	6	7.3	-0.3
2	7	7	8	7.3	8	8	8	8	0.7
3	7	8	7	7.3	8	7	8	7.6	0.3
4	6	5	9	6.6	8	10	9	9	2.4
5	11	11	10	10.6	10	9	10	9.6	-1
6	10	11	9	10	11	10	9	10	0
7	13	12	14	13	10	10	8	9.3	-3.7
8	12	13	10	11.6	12	9	10	10.3	-1.3
9	6	7	7	6.6	8	8	9	8.3	1.6
10	10	11	11	10.6	10	9	6	8.3	-2.3
**Mean**	**8.9**	**9.3**	**9.3**	**9.1**	**9.4**	**8.7**	**8.3**	**8.8**	**-0.3**
**SD**	**2.6**	**2.6**	**2.1**	**2.3**	**1.4**	**1.1**	**1.4**	**1**	**1**

B, Baseline; I, Intervention; SD, Standard Deviation. Standard Deviation. Values were expressed in numbers. Improvement was calculated as the difference between B and mean I Improvement was calculated as the difference between B and mean I (i.e., Improve = mean Intervention – mean Baseline). All negative values indicate no individual improvement.

Table S6. Purdue Pegboard Test – Bimanual subtest, groups data across all assessment points during baseline and intervention phases.

**Experimental Group**									
**Participant**	**B1**	**B2**	**B3**	**Mean**	**I1**	**I2**	**I3**	**Mean**	**Improve**
1	6	7	8	7	5	7	8	6.6	-0.4
2	8	7	9	8	10	10	10	10	2
3	8	10	10	9.3	11	11	11	11	1.6
4	9	10	11	10	10	11	12	11	1
5	10	10	10	10	12	12	13	12.3	2.3
6	10	10	11	10.3	12	13	13	12.6	2.3

7	10	11	11	10.6	12	13	11	12	1.4
8	12	9	12	11	10	13	14	12.3	1.3
9	10	10	10	10	11	11	15	12.3	2.3
10	7	8	8	7.6	10	11	12	11	3.3
**Mean**	**9**	**9.2**	**10**	**9.4**	**10.3**	**11.2**	**11.9**	**11.1**	**1.7**
**SD**	**1.7**	**1.3**	**1.8**	**1.3**	**2**	**1.8**	**2**	**1.7**	**0.9**
**Active Control Group**									
1	6	9	8	7.6	8	7	7	7.3	-0.3
2	6	7	5	6	8	7	6	7	1
3	5	7	8	6.6	9	9	9	9	2.4
4	9	9	11	9.6	8	12	10	10	0.4
5	10	10	10	10	9	10	10	9.6	-0.4
6	8	9	9	8.6	8	8	9	8.3	-0.3
7	11	10	10	10.3	10	10	8	9.3	-1
8	10	10	11	10.3	11	10	9	10	-0.3
9	6	7	8	7	9	9	7	8.3	1.3
10	10	9	10	9.6	10	10	11	10.3	0.6
**Mean**	**8.1**	**8.7**	**9**	**8.6**	**9**	**9.2**	**8.6**	**8.9**	**0.3**
**SD**	**2.1**	**1.2**	**1.8**	**1.6**	**1**	**1.5**	**1.5**	**1.1**	**1**

B, Baseline; I, Intervention; SD, Standard Deviation. Values were expressed in numbers. Improvement was calculated as the difference between B and mean I Improvement was calculated as the difference between B and mean I (i.e., Improve = mean Intervention – mean Baseline). All negative values indicate no individual improvement.

Table S7. Purdue Pegboard Test – Assembly subtest, groups data across all assessment points during baseline and intervention phases.

**Experimental Group**									
**Participant**	**B1**	**B2**	**B3**	**Mean**	**I1**	**I2**	**I3**	**Mean**	**Improve**
1	8	12	10	10	12	15	14	13.6	3.6
2	17	15	16	16	17	18	17	17.3	1.3
3	18	18	18	18	20	24	24	22.6	4.6
4	23	20	21	2.3	24	25	25	24.6	3.3
5	17	18	20	18.3	25	27	26	26	7.7
6	24	25	25	24.6	30	31	32	31	6.4

7	16	15	18	16.3	22	23	21	22	5.7
8	16	18	19	17.6	24	24	24	24	6.4
9	24	21	22	22.3	25	25	28	26	3.7
10	11	12	12	11.6	14	16	15	15	3.4
**Mean**	**17.4**	**17.4**	**18.1**	**17.6**	**21.3**	**22.8**	**22.6**	**22.3**	**4.7**
**SD**	**5.2**	**4**	**4.5**	**4.5**	**5.5**	**5**	**5.8**	**5.4**	**1.8**
				**Active Control Group**					
1	16	15	16	15.6	15	16	15	15.3	-0.3
2	12	13	12	12.3	12	15	14	13.6	1.3
3	16	13	18	15.6	15	15	16	15.3	-0.3
4	11	10	15	12	13	12	13	12.6	0.6
5	15	14	13	14	15	14	16	15	1
6	14	13	17	14.6	14	12	16	14	-0.6
7	24	23	21	22.6	21	21	24	22	-0.6
8	18	19	15	17.3	20	17	17	18	0.7
9	10	9	10	9.6	11	11	11	11	1.4
10	15	10	16	13.6	13	14	15	14	0.4
**Mean**	**15.1**	**13.9**	**15.3**	**14.7**	**14.9**	**14.7**	**15.7**	**15.1**	**0.4**
**SD**	**3.9**	**2**	**2.6**	**3.5**	**3.2**	**2.9**	**3.4**	**3**	**0.7**

B, Baseline; I, Intervention; SD, Standard Deviation. Values were expressed in numbers. Improvement was calculated as the difference between B and mean I (i.e., Improve = mean Intervention – mean Baseline). All negative values indicates no individual improvement.

Table S8. Purdue Pegboard Subtests - improvement between groups.

**Group**	**Unimanual Dominant**	**Unimanual Non-dominant**	**Bimanual**	**Assembly**
Experimental	2.03	1.5	1.73	4.6
SD	1.05	1.08	0.99	1.89
Active Control	-0.16	-0.36	0.33	0.33
SD	0.99	1.8	1	0.78

SD, Standard Deviation. Values were expressed in numbers. Improvement was calculated as the mean difference from each subtest between B and I (i.e., mean Intervention – mean Baseline). All negative values indicates no individual improvement.

Table S9. Timed 25-Foot Walk Test groups data across all assessment points during basely and intervention phases.

Experimental Group									
**Participant**	**B1**	**B2**	**B3**	**Mean**	**I1**	**I2**	**I3**	**Mean**	**Improve**
1	10.4	10.9	10.3	10.5	9.6	8.1	7.8	8.5	2
2	7.4	7.9	7.2	7.5	5.6	5.6	5.6	5.6	1.9
3	9.9	9.5	10.3	9.9	5.7	6.7	6.4	6.3	3.6
4	6.7	6.7	6.7	6.7	5.5	5.1	5.1	5.2	1.5
5	7.3	7.6	7.5	7.5	6.8	6	5.2	6	1.5
6	8.7	9.3	9.2	9	7.4	6.5	6.5	6.8	2.2
7	8.2	8.7	8.4	8.4	6.6	6.9	6.1	6.5	1.9
8	6.8	6.6	7.1	6.8	5.8	5	5.1	5.3	1.5
9	9.4	8.4	9.1	8.9	6.3	4.9	4.9	5.4	3.5
10	14.5	14.1	14.8	14.4	12.2	12.2	11.3	11.9	2.5
**Mean**	**8.9**	**8.9**	**9**	**9**	**7.1**	**6.7**	**6.4**	**6.7**	**2.3**
**SD**	**2.3**	**2.2**	**2.3**	**2.3**	**2.1**	**2.1**	**1.9**	**2**	**0.7**
**Active Control Group**									
1	11.9	11.7	12.4	12	12.4	12.7	12.4	12.5	-0.5
2	12	12	11.2	11.7	11.7	12.5	10.2	11.5	0.2
3	13.4	13.6	13.3	13.4	13.9	13.5	13.7	13.7	-0.3
4	7.7	8	7.6	7.8	8.3	7.7	7.5	7.8	0
5	6.7	6.7	6.6	6.6	6.5	7	6.7	6.7	0
6	8.4	8.6	8.2	8.4	8.6	8.1	8	8.2	0.2
7	5.5	6.2	5.8	5.8	5.6	6.1	5.5	5.7	0
8	6.6	6.7	6.6	6.6	6.6	6.7	6.5	6.6	0
9	10.5	10.7	10.3	10.5	10.5	10.9	10.4	10.6	0.1
10	11.6	11.8	11.6	11.6	11.9	12.1	11.3	11.8	-0.2
**Mean**	**9.4**	**9.6**	**9.3**	**9.4**	**9.6**	**9.7**	**9.2**	**9.5**	**0.1**
**SD**	**2.7**	**2.6**	**2.7**	**2.7**	**2.8**	**2.8**	**2.7**	**2.8**	**0.2**

B, Baseline; I, Intervention; SD, Standard Deviation. Values were expressed in numbers. Improvement was calculated as the difference between B and mean I Improvement was calculated as the difference between B and mean I (i.e., Improve = mean Intervention – mean Baseline). All negative values indicate no individual improvement.

Table S10. Six Spot Step Test groups data across all assessment points during baseline and intervention phases.

Experimental Group									
**Participant**	**B1**	**B2**	**B3**	**Mean**	**I1**	**I2**	**I3**	**Mean**	**Improve**
1	29.4	31.8	29.2	30.2	26	22.8	22.6	23.8	6.4
2	26.4	28.4	28.1	27.6	23.1	23	22.9	23	4.6
3	9.6	9.9	9.6	9.7	7.5	7.7	7.6	7.6	2.1
4	7.8	8.1	7.8	7.9	6.8	6.4	6.2	6.5	1.4
5	10.5	10.3	10.3	10.4	8.7	8.2	8.4	8.4	2
6	7	6.9	6.9	6.9	5.9	5.1	5.6	5.5	1.4
7	11.1	11.7	11.6	11.5	10.5	10.2	10.3	10.3	1.2
8	10.9	10.9	11.4	11.1	10	9.8	10	9.9	1.2
9	7.7	8	8.	7.9	6.8	6.6	6	6.5	1.4
10	10.1	10.8	10.3	10.4	9	8.8	8.4	8.7	1.7
**Mean**	**13**	**13.7**	**13.3**	**13.3**	**11.4**	**10.8**	**10.8**	**11**	**2.3**
**SD**	**7.9**	**8.8**	**8.2**	**8.3**	**7**	**6.5**	**6.4**	**6.6**	**1.7**
**Active Control Group**									
1	36.1	36.5	36.4	36.3	37.2	37.1	37.1	37.1	-0.8
2	19.5	20	19.7	19.7	19.8	19.6	20.1	19.8	0.1
3	8.7	8.4	8.6	8.5	8.9	8.4	8.7	8.7	-0.2
4	19.1	18.8	19.3	19	19.1	18.9	19.1	19	0
5	22.8	23.2	23	23	23.1	23.3	22.9	23.1	-0.1
6	7.4	7.6	7.4	7.5	7.8	7.5	7.6	7.6	-0.1
7	11.7	11.7	11.8	11.7	11.9	11.5	11.7	11.7	0
8	10.2	10.3	10.4	10.3	10.3	10.1	10.6	10.3	0
9	10.3	10.5	10.7	10.5	10.3	10.4	10.7	10.5	0
10	17.3	17.3	17.7	17.4	17.4	17.6	18	17.7	-0.3
**Mean**	**16.3**	**16.4**	**16.5**	**16.4**	**16.6**	**16.4**	**16.6**	**16.5**	**-0.1**
**SD**	**8.7**	**8.8**	**8.7**	**8.7**	**8.9**	**9**	**8.9**	**8.9**	**0.2**

B, Baseline; I, Intervention; SD, Standard Deviation. Values were expressed in numbers. Improvement was calculated as the difference between B and mean I Improvement was calculated as the difference between B and mean I (i.e., Improve = mean Intervention – mean Baseline). All negative values indicate no individual improvement.

Section S2_ANOVA analysis

Symbol Digit Modalities Test

Table S11. Symbol Digit Modalities Test – Within Subjects Effects.

Cases	F	*p*
Study Phases	326.20	< 0.001
Study Phases ✻ Group	75.25	< 0.001

✻, Interaction between the two variables.

Table S12. Symbol Digit Modalities Test – Post Hoc Comparisons – Study Phases.

		Mean Difference	SE	t	p_{bonf}
Baseline	Intervention	-4.23	0.23	-18.06	< 0.001

Table S13. Symbol Digit Modalities Test – Post Hoc Comparisons – Group ✻ Study Phases.

		Mean Difference	SE	t	p_{bonf}
Experimental, Baseline	Active Control, Baseline	0.06	6.07	0.01	1.000
	Experimental, Intervention	-6.26	0.33	-18.9	< 0.001
	Active Control, Intervention	-2.13	6.1	-0.35	1.000
Active Control, Baseline	Experimental, Intervention	-6.33	6.1	-1.03	1.000
	Active Control, Intervention	-2.2	0.33	-6.63	< 0.001
Experimental, Intervention	Active Control, Intervention	4.13	6.14	0.67	1.000

Results are averaged over the levels of: Assessments Points.

Table S14. Symbol Digit Modalities Test – *t*-test – Improvement difference between groups.

	Experimental Group	Active Control Group

Mean	6.2	2.2
Observations	10	10
df	18	
t statistic	8.6	
p - value	< 0.05	

Medical Outcomes Study Questionnaire Short Form 36 Health Survey

Table S15. Medical Outcomes Study Questionnaire Short Form 36 Health Survey – Within Subjects Effects.

Cases	F	*p*
Study Phases	4.86	0.005
Study Phases ✻ Group	7	< 0.001

✻, Interaction between the two variables.

Table S16. Medical Outcomes Study Questionnaire Short Form 36 Health Survey – Post Hoc Comparisons – Study Phases.

		Mean Difference	SE	t	p_{bonf}
Baseline	I1	-2.8	0.39	-7.01	< 0.001
	I2	-3.55	0.59	-5.93	< 0.001
	I3	-3.75	1.46	-2.56	0.117
I1	I2	-0.75	0.49	-1.5	0.897
	I3	-0.95	1.53	-0.61	1.000
I2	I3	-0.2	1.46	-0.13	1.000

Table S17. Medical Outcomes Study Questionnaire Short Form 36 Health Survey – Post Hoc Comparisons - Group ✻ Study Phases.

		Mean Difference	SE	t	p_{bonf}
Experimental, Baseline	Active Control, Baseline	3.1	3.27	0.94	1.000
	Experimental, I1	-5.1	0.56	-9.03	< 0.001
	Active Control, I1	2.6	3.32	0.78	1.000
	Experimental, I2	-6.5	0.84	-7.68	< 0.001
	Active Control, I2	2.5	3.36	0.74	1.000
	Experimental, I3	-8.8	2.06	-4.25	0.013
	Active Control, I3	4.4	3.08	1.42	1.000

Active Control, Baseline	Experimental, I1	-8.2	3.32	-2.46	0.672
	Active Control, I1	-0.5	0.56	-0.88	1.000
	Experimental, I2	-9.6	3.36	-2.85	0.294
	Active Control, I2	-0.6	0.84	-0.71	1.000
	Experimental, I3	-11.9	3.08	-3.86	0.032
	Active Control, I3	1.3	2.06	0.62	1.000
Experimental, I1	Active Control, I1	7.7	3.38	2.27	0.99
	Experimental, I2	-1.4	0.7	-1.98	1.000
	Active Control, I2	7.6	3.41	2.22	1.000
	Experimental, I3	-3.7	2.17	-1.7	1.000
	Active, Control, I3	9.5	3.1	3.02	0.2
Active Control, I1	Experimental, I2	-9.1	3.4	-2.66	0.44
	Active Control, I2	-0.1	0.7	-0.14	1.000
	Experimental, I3	-11.4	3.14	-3.62	0.054
	Active Control, I3	1.8	2.17	0.82	1.000
Experimental, I2	Active Control, I2	9	3.45	2.6	0.49
	Experimental, I3	-2.3	2.07	-1.11	1.000
	Active Control, I3	10.9	3.18	3.43	0.084
Active Control, I2	Experimental, I3	-11.3	3.18	-3.55	0.063
	Active Control, I3	1.9	2.07	0.91	1.000
Experimental, I3	Active Control, I3	13.2	2.88	4.58	0.006

I, Intervention.

Table S18. Medical Outcomes Study Questionnaire Short Form 36 Health Survey – *t*-test – Improvement difference between groups.

	Experimental Group	Active Control Group
Mean	6.8	0.98
Observations	10	10
df	18	
t statistic	7.03	
p - value	< 0.05	

Modified Fatigue Impact Scale

Table S19. Modified Fatigue Impact Scale – Within Subjects Effects.

Cases	F	*p*
Study Phases	11.18	< 0.001
Study Phases ✻ Group	13.88	< 0.001

✻, Interaction between the two variables.

Table S20. Modified Fatigue Impact Scale – Post Hoc Comparisons – Study Phases.

		Mean Difference	SE	t	p_{bonf}
Baseline	I1	3.700	1.143	3.238	0.027
	I2	5.250	1.343	3.909	0.006
	I3	6.200	1.606	3.860	0.007
I1	I2	1.550	0.593	2.615	0.105
	I3	2.500	1.170	2.137	0.280
I2	I3	0.950	0.754	1.259	1.000

I, Intervention. Results are averaged over the levels of: Group.

Table S21. Modified Fatigue Impact Scale – Post Hoc Comparisons – Group ✻ Study Phases.

		Mean Difference	SE	t	p_{bonf}
Experimental, Baseline	Active Control, Baseline	-7.4	7.88	-0.9	1.000
	Experimental, I1	7.7	1.61	4.76	0.004
	Active Control, I1	-7.7	7.78	-0.1	1.000
	Experimental, I2	11.1	1.89	5.84	< 0.001
	Active Control, I2	-8	7.91	-1	1.000
	Experimental, I3	13.1	2.27	5.76	< 0.001
	Active Control, I3	-8.1	8.23	-0.1	1.000
Active Control, Baseline	Experimental, I1	15.1	7.78	1.94	1.000
	Active Control, I1	-0.3	1.61	-0.2	1.000
	Experimental, I2	18.5	7.91	2.33	0.873

	Active Control, I2	-0.6	1.89	-0.3	1.000
	Experimental, I3	20.5	8.23	2.49	0.637
	Active Control, I3	-0.7	2.27	-0.3	1.000
Experimental, I1	Active Control, I1	-15.4	7.68	-2	1.000
	Experimental, I2	3.4	0.83	4.05	0.021
	Active Control, I2	-15.7	7.81	-2	1.000
	Experimental, I3	5.4	1.65	3.26	0.121
	Active Control, I3	-15.8	8.13	-1.9	1.000
Active Control, I1	Experimental, I2	18.8	7.81	2.4	0.759
	Active Control, I2	-0.3	0.83	-0.3	1.000
	Experimental, I3	20.8	8.13	2.55	0.555
	Active Control, I3	-0.4	1.65	-0.2	1.000
Experimental, I2	Active Control, I2	-19.1	7.94	-2.4	0.761
	Experimental, I3	2	1.06	1.87	1.000
	Active Control, I3	-19.2	8.26	-2.3	0.896
Active Control, I2	Experimental, I3	21.1	8.26	2.55	0.558
	Active Control, I3	-0.1	1.06	-0.1	1.000
Experimental, I3	Active Control, I3	-21.2	8.56	-2.4	0.658

I, Intervention.

Table S22. Modified Fatigue Impact Scale – *t*-test – Improvement difference between groups.

	Experimental Group	Active Control Group
Mean	10.6	0.29
Observations	10	10
df	18	
t statistic	4.1	
p - value	< 0.05	

Purdue Pegboard Test

Table S23. Purdue Pegboard Test – Within Subjects Effects.

Cases	F	p
Study Phases	45.95	< 0.001
Study Phases ✻ Group	43.53	< 0.001

Subtests	100.23	< 0.001
Subtests ✻ Group	4.51	0.007
Subtests ✻ Subtests ✻Group	6.6	< 0.001

✻, Interaction between the two variables.

Table S24. Purdue Pegboard test – Post Hoc Comparisons – Study Phases.

		Mean Difference	SE	t	p_{bonf}
Baseline	Intervention	-1.25	0.18	-6.77	< 0.001

Results are averaged over the levels of: Group, Subtests, Assessment Points.

Table S25. Purdue Pegboard test – Post Hoc Comparisons – Subtests.

		Mean Difference	SE	t	p_{bonf}
Unimanual Dominant	Unimanual Non-Dominant	1.93	0.23	8.40	< 0.001
	Bimanual	2.43	0.29	8.35	< 0.001
	Assembly	-5.48	0.62	-8.71	< 0.001
Unimanual Non-Dominant	Bimanual	0.5	0.23	2.15	0.270
	Assembly	-7.41	0.63	-11.62	< 0.001
Bimanual	Assembly	-7.91	0.76	-10.41	< 0.001

Results are averaged over the levels of: Group, Assessment Points, Study Phases.

Table S26. Purdue Pegboard test – Post Hoc Comparisons – Group ✻ Study Phases ✻ Subtests.

		Mean Difference	SE	t	p_{bonf}
Experimental, Baseline, Unimanual Dominant	Active Control, Baseline, Unimanual Dominant	1.5	0.94	1.58	1.000
	Experimental, Intervention, Unimanual Dominant	-2.03	0.32	-6.25	< 0.001
	Active Control, Intervention, Unimanual Dominant	1.66	0.9	1.84	1.000
	Experimental, Baseline, Unimanual Non-Dominant	1.93	0.35	5.39	0.005

	Active Control, Baseline, Unimanual Non-Dominant	3.06	0.96	3.17	0.623
	Experimental, Intervention, Unimanual Non-Dominant	0.43	0.5	0.86	1.000
	Active Control, Intervention, Unimanual Non-Dominant	3.43	0.86	3.95	0.111
	Experimental, Baseline, Bimanual	2.83	0.47	5.92	0.002
	Active Control, Baseline, Bimanual	3.63	0.82	4.42	0.04
	Experimental, Intervention, Bimanual	1.1	0.51	2.13	1.000
	Active Control, Intervention, Bimanual	3.3	0.82	4.02	0.096
	Experimental, Baseline, Assembly	-5.4	0.838	-6.44	< 0.001
	Active Control, Baseline, Assembly	-2.53	1.44	-1.75	1.000
	Experimental, Intervention, Assembly	-10	0.94	-10.6	< 0.001
	Active Control, Intervention, Assembly	-2.86	1.54	-1.85	1.000
Active Control, Baseline, Unimanual Dominant	Experimental, Intervention, Unimanual Dominant	-3.53	0.9	-3.91	0.121
	Active Control, Intervention, Unimanual Dominant	0.16	0.32	0.51	1.000
	Experimental, Baseline, Unimanual Non-Dominant	0.43	0.96	0.44	1.000
	Active, Control, Baseline, Unimanual Non-Dominant	1.56	0.35	4.37	0.044
	Experimental, Intervention, Unimanual Non-Dominant	-1.06	0.86	-1.22	1.000
	Active Control, Intervention, Unimanual Non-Dominant	1.93	0.5	3.84	0.143
	Experimental, Baseline, Bimanual	1.33	0.82	1.62	1.000
	Active Control, Baseline, Bimanual	2.13	0.47	4.45	0.036
	Experimental, Intervention, Bimanual	-0.4	0.82	-0.48	1.000

	Active Control, Intervention, Bimanual	1.8	0.51	3.5	0.307
	Experimental, Baseline, Assembly	-6.9	1.44	-4.76	0.018
	Active Control, Baseline, Assembly	-4.03	0.83	-4.81	0.017
	Experimental, Intervention, Assembly	-11.5	1.54	-7.45	< 0.001
	Active Control, Intervention, Assembly	-4.36	0.94	-4.63	0.025
Experimental, Intervention, Unimanual Dominant	Active Control, Intervention, Unimanual Dominant	3.7	0.85	4.33	0.049
	Experimental, Baseline, Unimanual Non-Dominant	3.96	0.46	8.60	< 0.001
	Active Control, Baseline, Uniman-ual Non-Dominant	5.1	0.92	5.54	0.004
	Experimental, Intervention, Unimanual Non-Dominant	2.46	0.39	6.24	< 0.001
	Active, Control, Intervention, Unimanual Non-Dominant	5.46	0.81	6.67	< 0.001
	Experimental, Baseline, Bimanual	4.86	0.46	10.54	< 0.001
	Active Control, Baseline, Bimanual	5.66	0.77	7.35	< 0.001
	Experimental, Intervention, Bimanual	3.13	0.42	7.44	< 0.001
	Active Control, Intervention, Bimanual	5.33	0.77	6.93	< 0.001
	Experimental, Baseline, Assembly	-3.37	0.92	-3.63	0.226
	Active Control, Baseline, Assembly	-0.5	1.41	-0.35	1.000
	Experimental, Intervention, Assembly	-7.96	1.01	-7.83	< 0.001
	Active Control, Intervention, Assembly	-0.83	1.51	-0.55	1.000
Active Control, Intervention, Unimanual Dominant	Experimental, Baseline, Unimanual Non-Dominant	0.26	0.92	0.29	1.000
	Active Control, Baseline,	1.4	0.46	3.03	0.853

	Unimanual Non-Dominant				
	Experimental, Intervention, Unimanual Non-Dominant	-1.23	0.82	-1.5	1.000
	Active Control, Intervention, Unimanual Non-Dominant	1.76	0.39	4.47	0.035
	Experimental, Baseline, Bimanual	1.16	0.77	1.51	1.000
	Active Control, Baseline, Bimanual	1.96	0.46	4.26	0.057
	Experimental, Intervention, Bimanual	-0.56	0.77	-0.73	1.000
	Active Control, Intervention, Bimanual	1.63	0.42	3.88	0.132
	Experimental, Baseline, Assembly	-7.067	1.418	-4.98	0.012
	Active Control, Baseline, Assembly	-4.2	0.92	-4.54	0.031
	Experimental, Intervention, Assembly	-11.66	1.51	-7.7	< 0.001
	Active, Control, Intervention, Assembly	-4.53	1.01	-4.45	0.036
Experimental, Baseline, Unimanual Non-Dominant	Active Control, Baseline, Unimanual Non-Dominant	1.13	0.98	1.15	1.000
	Experimental, Intervention, Unimanual Non-Dominant	-1.5	0.47	-3.19	0.604
	Active Control, Intervention, Unimanual Non-Dominant	1.5	0.88	1.69	1.000
	Experimental, Baseline, Bimanual	0.9	0.43	2.09	1.000
	Active Control, Baseline, Bimanual	1.7	0.84	2.01	1.000
	Experimental, Intervention, Bimanual	-0.83	0.54	-1.54	1.000
	Active, Control, Intervention, Bimanual	1.36	0.84	1.62	1.000
	Experimental, Baseline, Assembly	-7.33	0.87	-8.41	< 0.001
	Active Control, Baseline, Assembly	-4.46	1.46	-3.06	0.805
	Experimental, Intervention, Assembly	-11.93	0.9	-13.24	< 0.001

	Active Control, Intervention, Assembly	-4.8	1.55	-3.09	0.758
Active Control, Baseline, Unimanual Non-Dominant	Experimental, Intervention, Unimanual Non-Dominant	-2.63	0.88	-2.96	0.991
	Active Control, Intervention, Unimanual Non-Dominant	0.36	0.47	0.78	1.000
	Experimental, Baseline, Bimanual	-0.23	0.84	-0.27	1.000
	Active Control, Baseline, Bimanual	0.56	0.43	1.31	1.000
	Experimental, Intervention, Bimanual	-1.96	0.84	-2.33	1.000
	Active Control, Intervention, Bimanual	0.23	0.54	0.43	1.000
	Experimental, Baseline, Assembly	-8.46	1.46	-5.8	0.002
	Active Control, Baseline, Assembly	-5.6	0.87	-6.42	< 0.001
	Experimental, Intervention, Assembly	-13.06	1.55	-8.41	< 0.001
	Active, Control, Intervention, Assembly	-5.93	0.9	-6.58	< 0.001
Experimental, Intervention, Unimanual Non-Dominant	Active Control, Intervention , Unimanual Non-Dominant	3	0.78	3.83	0.144
	Experimental, Baseline, Bimanual	2.4	0.38	6.18	< 0.001
	Active Control, Baseline, Bimanual	3.2	0.73	4.38	0.043
	Experimental, Intervention, Bimanual	0.66	0.35	1.88	1.000
	Active Control, Intervention, Bimanual	2.86	0.73	3.93	0.117
	Experimental, Baseline, Assembly	-5.83	1.02	-5.71	0.002
	Active Control, Baseline, Assembly	-2.96	1.39	-2.12	1.000
	Experimental, Intervention, Assembly	-10.43	1.03	-10.07	< 0.001
	Active Control, Intervention, Assembly	-3.3	1.49	-2.2	1.000
Active Control, Intervention,	Experimental, Baseline, Bimanual	-0.6	0.73	-0.82	1.000

Unimanual Non-Dominant					
	Active Control, Baseline, Bimanual	0.2	0.38	0.51	1.000
	Experimental, Intervention, Bimanual	-2.33	0.73	-3.2	0.594
	Active Control, Intervention, Bimanual	-0.13	0.35	-0.37	1.000
	Experimental, Baseline, Assembly	-8.83	1.39	-6.32	< 0.001
	Active Control, Baseline, Assembly	-5.96	1.02	-5.84	0.002
	Experimental, Intervention, Assembly	-13.43	1.49	-8.98	< 0.001
	Active Control, Intervention, Assembly	-6.3	1.03	-6.08	0.001
Experimental, Baseline, Bimanual	Active Control, Baseline, Bimanual	0.8	0.67	1.18	1.000
	Experimental, Intervention, Bimanual	-1.73	0.31	-5.48	0.004
	Active Control, Intervention, Bimanual	0.46	0.67	0.69	1.000
	Experimental, Baseline, Assembly	-8.23	1.06	-7.72	< 0.001
	Active Control, Baseline, Assembly	-5.36	1.37	-3.92	0.120
	Experimental, Intervention, Assembly	-12.83	1.13	-11.3	< 0.001
	Active Control, Intervention, Assembly	-5.7	1.47	-3.88	0.132
Active Control, Baseline, Bimanual	Experimental, Intervention, Bimanual	-2.53	0.67	-3.76	0.172
	Active Control, Intervention, Bimanual	-0.33	0.31	-1.05	1.000
	Experimental, Baseline, Assembly	-9.03	1.37	-6.6	< 0.001
	Active Control, Baseline, Assembly	-6.16	1.06	-5.78	0.002
	Experimental, Intervention, Assembly	-13.63	1.47	-9.28	< 0.001
	Active Control, Intervention,	-6.5	1.13	-5.75	0.002

	Assembly				
Experimental, Intervention, Bimanual	Active Control, Intervention, Bimanual	2.2	0.67	3.27	0.507
	Experimental, Baseline, Assembly	-6.5	1.1	-5.91	0.002
	Active Control, Baseline, Assembly	-3.63	1.36	-2.65	1.000
	Experimental, Intervention, Assembly	-11.1	1.14	-9.68	< 0.001
	Active Control, Intervention, Assembly	-3.96	1.47	-2.7	1.000
Active Control, Intervention, Bimanual	Experimental, Baseline, Assembly	-8.7	1.36	-6.36	< 0.001
	Active Control, Baseline, Assembly	-5.83	1.1	-5.3	0.006
	Experimental, Intervention, Assembly	-13.3	1.47	-9.05	< 0.001
	Active, Control, Intervention, Assembly	-6.16	1.14	-5.38	0.005
Experimental, Baseline, Assembly	Active Control, Baseline, Assembly	2.86	1.81	1.58	1.000
	Experimental, Intervention, Assembly	-4.6	0.45	-10	< 0.001
	Active Control, Intervention, Assembly	2.53	1.89	1.34	1.000
Active Control, Baseline, Assem-bly	Experimental, Intervention, Assembly	-7.46	1.89	-3.94	0.113
	Active Control, Intervention, Assembly	-0.33	0.45	-0.72	1.000
Experimental, Intervention, Assembly	Active Control, Intervention, Assembly	7.13	1.96	3.63	0.230

Results are averaged over the levels of: Assessment Points.

Table S27. Purdue Pegboard Subtests improvement – Within Subjects Effects.

Cases	F	*p*

Purdue Pegboard Subtests	11.51	< 0.001
Purdue Pegboard Subtests ✻ Group	6.6	< 0.001

✻, Interaction between the two variables.

Table S28. Purdue Pegboard Subtests improvement – Post Hoc Comparisons – Group ✻ Subtests.

		Mean Difference	SE	t	p_{bonf}
Experimental, Unimanual Dominant	Active Control, Unimanual Dominant	2.2	0.46	4.7	0.004
	Experimental, Unimanual Non-Dominant	0.53	0.38	1.4	1.000
	Active Control, Unimanual Non-Dominant	2.4	0.57	4.2	0.015
	Experimental, Bimanual	0.3	0.36	0.82	1.000
	Active Control, Bimanual	1.700	0.453	3.75	0.041
	Experimental, Assembly	-2.567	0.554	-4.6	0.006
	Active Control, Assembly	1.700	0.562	3.02	0.203
Active Control, Unimanual Dominant	Experimental, Unimanual Non-Dominant	-1.66	0.57	-2.9	0.258
	Active Control, Unimanual Non-Dominant	0.2	0.38	0.52	1.000
	Experimental, Bimanual	-1.9	0.45	-4.2	0.015
	Active Control, Bimanual	-0.5	0.36	-1.3	1.000
	Experimental, Assembly	-4.76	0.562	-8.4	< 0.001
	Active Control, Assembly	-0.5	0.55	-0.9	1.000
Experimental, Unimanual Non-Dominant	Active Control, Unimanual Non-Dominant	1.86	0.66	2.81	0.324
	Experimental, Bimanual	-0.23	0.43	-0.5	1.000
	Active Control, Bimanual	1.16	0.566	2.06	1.000
	Experimental, Assembly	-3.1	0.63	-4.8	0.004
	Active Control, Assembly	1.16	0.656	1.77	1.000
Active Control, Unimanual Non-Dominant	Experimental, Bimanual	-2.1	0.56	-3.7	0.045
	Active Control, Bimanual	-0.7	0.43	-1.6	1.000

	Experimental, Assembly	-4.96	0.65	-7.5	< 0.001
	Active Control, Assembly	-0.7	0.63	-1	1.000
Experimental, Bimanual	Active Control, Bimanual	1.4	0.44	3.13	0.160
	Experimental, Assembly	-2.86	0.52	-5.4	< 0.001
	Active Control, Assembly	1.4	0.55	2.51	0.603
Active Control, Bimanual	Experimental, Assembly	-4.26	0.55	-7.6	< 0.001
	Active Control, Assembly	$1.\times10^{-10}$	0.52	2×10^{-10}	1.000
Experimental, Assembly	Active Control, Assembly	4.26	0.64	6.58	< 0.001

Timed 25-Foot Walk Test

Table S29. Timed 25-Foot Walk Test – Within Subjects Effects.

Cases	F	*p*
Study Phases	69.87	< 0.001
Study Phases ✻ Group	76.68	< 0.001

✻, Interaction between the two variables.

Table S30. Timed 25-Foot Walk Test – Post Hoc Comparisons – Study phases.

		Mean Difference	SE	t	p_{bonf}
Baseline	Intervention	1.08	0.13	8.36	< 0.001

Results are averaged over the levels of: Group, Assessments Points.

Table S31. Timed 25-Foot Walk Test – Post Hoc Comparisons – Group ✻ Study Phases.

		Mean Difference	SE	t	p_{bonf}
Experimental, Baseline	Active Control, Baseline	-0.48	1.12	-0.42	1.000
	Experimental, Intervention	2.22	0.18	12.1	< 0.001
	Active Control, Intervention	-0.53	1.11	-0.4	1.000
Active Control, Baseline	Experimental, Intervention	2.7	1.11	2.42	0.156
	Active Control, Intervention	-0.05	0.18	-0.2	1.000

Experimental, Intervention	Active Control, Intervention	-2.75	1.10	-2.4	0.135

Results are averaged over the levels of Assessments Points.

Table S32. Timed 25-Foot Walk Test – *t*-test – Improvement difference between groups.

	**Experimental Group**	**Active Control Group**
Mean	2.2	-0.05
Observations	10	10
df	18	
t statistic	8.5	
p - value	< 0.05	

<u>Six Spot Step Test</u>

Table S33. Six Spot Step Test – Within Subjects Effects.

**Cases**	**F**	***p***
Study Phases	15.43	< 0.001
Study Phases ✻ Group	19.67	< 0.001

✻, Interaction between the two variables.

Table S34. Six Spot Step Test – Post Hoc Comparisons – Study – Study phases.

		**Mean Difference**	**SE**	**t**	p_{bonf}
Baseline	Intervention	1.09	0.28	3.93	< 0.001

Results are averaged over the levels of: Group, Assessments Points.

Table S35. Six Spot Step Test – Post Hoc Comparisons - Group ✻ Study Phases.

		**Mean Difference**	**SE**	**t**	p_{bonf}
Experimental, Baseline	Active Control, Baseline	-3.05	3.83	-0.8	1.000
	Experimental, Intervention	2.33	0.39	5.91	< 0.001

	Active Control, Intervention	-3.19	3.69	-0.8	1.000
Active Control, Baseline	Experimental, Intervention	5.39	3.69	1.46	0.967
	Active Control, Intervention	-0.14	0.39	-0.3	1.000
Experimental, Intervention	Active Control, Intervention	-5.53	3.54	-1.5	0.814

Results are averaged over the levels of: Assessment Points.

Table S36. Six Spot Step Test – *t*-test – Improvement difference between groups.

	**Experimental Group**	**Active Control Group**
Mean	2.3	-0.1
Observations	10	10
df	9	
t statistic	4	
p - value	< 0.05	
